# Multiscale heterogeneity in gastric adenocarcinoma evolution is an obstacle to precision medicine

**DOI:** 10.1186/s13073-021-00975-y

**Published:** 2021-11-08

**Authors:** Christoph Röcken, Anu Amallraja, Christine Halske, Luka Opasic, Arne Traulsen, Hans-Michael Behrens, Sandra Krüger, Anne Liu, Jochen Haag, Jan-Hendrik Egberts, Philip Rosenstiel, Tobias Meißner

**Affiliations:** 1 Department of Pathology, Christian-Albrechts-University, University Hospital Schleswig-Holstein (UKSH), Campus Kiel, Arnold-Heller-Str. 3, Haus U33, D-24105 Kiel, Germany; 2 Institute for Clinical Molecular Biology, Christian-Albrechts-University, 24105 Kiel, Germany; 3 Department of Molecular and Experimental Medicine, Avera Cancer Institute, Sioux Falls, USA; 4 Department of Evolutionary Theory, Max Planck Institute for Evolutionary Biology, Plön, Germany; 5 Department of General Surgery, Visceral, Thoracic, Transplantation and Pediatric Surgery, University Hospital Schleswig-Holstein (UKSH), Campus Kiel, Kiel, Germany

**Keywords:** Gastric cancer, Intratumoral heterogeneity, Evolution, SMAD4, TP53

## Abstract

**Background:**

Cancer is a somatic evolutionary disease and adenocarcinomas of the stomach and gastroesophageal junction (GC) may serve as a two-dimensional model of cancer expansion, in which tumor subclones are not evenly mixed during tumor progression but rather spatially separated and diversified. We hypothesize that precision medicine efforts are compromised when clinical decisions are based on a single-sample analysis, which ignores the mechanisms of cancer evolution and resulting intratumoral heterogeneity. Using multiregional whole-exome sequencing, we investigated the effect of somatic evolution on intratumoral heterogeneity aiming to shed light on the evolutionary biology of GC.

**Methods:**

The study comprised a prospective discovery cohort of 9 and a validation cohort of 463 GCs. Multiregional whole-exome sequencing was performed using samples form 45 primary tumors and 3 lymph node metastases (range 3–10 tumor samples/patient) of the discovery cohort.

**Results:**

In total, the discovery cohort harbored 16,537 non-synonymous mutations. Intratumoral heterogeneity of somatic mutations and copy number variants were present in all tumors of the discovery cohort. Of the non-synonymous mutations, 53–91% were not present in each patient’s sample; 399 genes harbored 2–4 different non-synonymous mutations in the same patient; 175 genes showed copy number variations, the majority being heterogeneous, including CD274 (PD-L1). Multi-sample tree-based analyses provided evidence for branched evolution being most complex in a microsatellite instable GC. The analysis of the mode of evolution showed a high degree of heterogeneity in deviation from neutrality within each tumor. We found evidence of parallel evolution and evolutionary trajectories: different mutations of *SMAD4* aligned with different subclones and were found only in *TP53* mutant GCs.

**Conclusions:**

Neutral and non-neutral somatic evolution shape the mutational landscape in GC along its lateral expansions. It leads to complex spatial intratumoral heterogeneity, where lymph node metastases may stem from different areas of the primary tumor, synchronously. Our findings may have profound effects on future patient management. They illustrate the risk of mis-interpreting tumor genetics based on single-sample analysis and open new avenues for an evolutionary classification of GC, i.e., the discovery of distinct evolutionary trajectories which can be utilized for precision medicine.

**Supplementary Information:**

The online version contains supplementary material available at 10.1186/s13073-021-00975-y.

## Background

Gastric cancer (GC) is the fifth most common cancer in the world [1]. In Western countries, the prognosis is dismal due to diagnoses in advanced disease stages often limiting therapeutic options. Compared with non-small cell lung cancer, targeted palliative therapeutic options are still limited in GC, though they demonstrated significant efficacy in a more recent umbrella trial [2]. An integrative genomic analysis of the *Cancer Genome Atlas Research Network* proposed a roadmap for patient stratification and trials of targeted therapies by categorizing GC into four subtypes: Epstein-Barr virus-associated (EBV), microsatellite unstable (MSI), chromosomal unstable (CIN), and genomically stable (GS) GC [3]. Various validation studies lead to the identification of a marked intratumoral heterogeneity, which stands to compromise the development and usage of targeted therapies in GC [4–9]. These observations suggest that processes of somatic evolution are an important part of GC biology. Different tumor subclones coexist and contribute to genetic and phenotypic diversity. However, the modes of evolution operative in GC are largely unknown. Somatic evolution, i.e., the temporal/phylogenetic order and spatial distribution of mutations, is an important determinant of intratumoral heterogeneity. In a reductionist view, GC may serve as a two-dimensional model of cancer expansion, which can be used to shed light on its evolutionary biology, and to unravel obstacles and chances for precision medicine. Using multiregional whole-exome sequencing of primary GCs, we confirmed the presence of a substantial intratumoral and intermetastatic heterogeneity, which applies to variant allele frequencies, the type of single-nucleotide variation, and copy number variation. We found evidence of a neutral and non-neutral cancer expansion model in GC and of evolutionary trajectories leading to parallel evolution, which may pave the way to an “evolutionary classification” of GC.

## Methods

### Study population and histology

#### Discovery cohort (Table 1)

Between 2016 and 2017, we prospectively enrolled nine patients with an adenocarcinoma of the stomach or esophagogastric junction into the discovery cohort at the University Hospital Schleswig-Holstein, Campus Kiel. All patients were Caucasian patients from Northern Germany treated in a single center. The inclusion criteria were appropriate size of the primary tumor (diameter > 3 cm) to enable multiregional tissue sampling without compromising the surgical pathological evaluation of the resection specimen. Immediately after the tumor was resected, the specimens were delivered on ice to the Department of Pathology. Depending on the size of the primary tumor, between 3 and 6 samples were punched out of the primary tumor using a core needle biopsy and frozen at − 80 °C until further use. Macroscopic pictures were taken from the surgical resection specimens before and after tissue sampling in order to facilitate anatomical reconstruction of the sampling procedure (Additional file 1: Figure S1). A total of 45 samples were obtained from the primary tumors. In a single case, three samples were collected from three separate lymph node metastases. Finally, 48 tumor samples and nine tissue samples of the corresponding non-neoplastic stomach mucosa, i.e., 57 tissue samples in total, were forwarded to whole-exome sequencing (Table 1).

**Table 1:** Clinicopathological patient characteristics of the gastric cancer test cohort consisting of nine men (mean age 68 years; range 50–85 years)

Case	Case #1		Case #2		Case #3		Case #4		Case #5		Case #6		Case #7		Case #8		Case #9	
**Localization**	Cardia		Cardia		Antrum/Corpus		Antrum		Fundus		Cardia		Cardia		Cardia		Antrum	
**Neoadjuvant treatment**	Yes		Yes		No		No		No		No		Yes		No		Yes	
**Laurén phenotype**	Intestinal		Mixed		Mixed		Mixed		Diffuse		Intestinal		Intestinal		Mixed		Diffuse	
**Tumor size [cm]**	3.8		4.4		12.9		4.0		4.8		4.1		6.7		6.2		3.7	
**Epstein-Barr virus status**	Negative		Negative		Negative		Negative		Negative		Negative		Negative		Negative		Negative	
**Microsatellite status**	MSS		MSS		MSS		MSS		MSS		**MSI**		MSS		MSS		MSS	
**HER2 status**	Negative		Negative		Positive		Positive		Negative		Negative		Negative		Negative		Negative	
**pT category**	ypT3		ypT3		pT3		pT3		pT3		pT3		ypT3		pT2		ypT3	
**pN category**	ypN1 (1/29)		ypN1 (2/51)		pN3a (13/17)		pN2 (3/19)		pN3b (28/46)		pN0 (0/19)		ypN0 (0/12)		pN0 (0/25)		ypN2 (4/22)	
**pM category**	X		X		X		X		X		X		X		X		X	
**Grading**	n.a.		n.a.		G3		G3		G3		G2		n.a.		n.a.		n.a.	
**Number of tumor samples sequenced**	4		3		6		5		10		5		4		6		5	
**Non-synonymous mutations (total valid)**	184		373		714		348		425		3111		181		369		242	
**Copy number variations (number of genes)**	6		22		65		11		98		3		5		1		0	
**Tumor sample**	**Number/percentage of non-synonymous mutations present only in a single sample**																	
1 sample	71	(38.6%)	159	(42.6%)	350	(49.0%)	133	(38.2%)	211	(49.6%)	1262	(40.6%)	112	(61.9%)	235	(63.7%)	140	(57.8%)
	**Number/percentage of same non-synonymous mutations present in ≥ 2 samples**																	
2 samples	25	(13.6%)	59	(15.8%)	78	(10.9%)	15	(4.3%)	29	(6.8%)	360	(11.6%)	24	(13.3%)	25	(6.8%)	30	(12.4%)
3 samples	58	(31.5%)	155	(41.6%)	31	(4.3%)	11	(3.2%)	27	(6.4%)	180	(5.8%)	18	(9.9%)	9	(2.4%)	23	(9.5%)
4 samples	30	(16.3%)			8	(1.1%)	26	(7.5%)	17	(4.0%)	148	(4.8%)	27	(14.9%)	14	(3.8%)	28	(11.6%)
5 samples					25	(3.5%)	163	(46.8%)	13	(3.1%)	1161	(37.3%)			17	(4.6%)	21	(8.7%)
6 samples					222	(31.1%)			18	(4.2%)					69	(18.7%)		
7 samples									24	(5.6%)								
8 samples									15	(3.5%)								
9 samples									25	(5.9%)								
10 samples									46	(10.8%)								
**Per patient**	**Clonality**																	
Clonal (non-synonymous)	2	(2.0%)	80	(50.0%)	62	(23.9%)	1	(0.9%)	1	(1.3%)	0	(0%)	0	(0%)	0	(0%)	0	(0%)
Subclonal (non-synonymous)	98	(98.0%)	80	(50.0%)	197	(76.1%)	110	(98.2%)	76	(98.7%)	1083	(100%)	48	(100%)	81	(100%)	106	(100%)
Not assessable (non-synonymous)	0	(0%)	0	(0%)	0	(0%)	1	(0.9%)	0	(0%)	0	(0%)	0	(0%)	0	(0%)	0	(0%)
Total (non-synonymous)	100		160		259		112		77		1083		48		81		106	
Clonal (synonymous)	1	(6.0%)	32	(15.9%)	26	(10.1%)	2	(1.3%)	1	(5.7%)	0	(0%)	1	(0.4%)	0	(0%)	0	(0%)
Subclonal (synonymous)	159	(94.0%)	168	(83.6%)	231	(89.9%)	157	(98.7%)	174	(99.4%)	825	(97.6%)	229	(99.6%)	168	(100%)	257	(99.6%)
Not assessable (synonymous)	0	(0%)	1	(4.9%)	0	(0%)	0	(0%)	0	(0%)	2	(2.4%)	0	(0%)	0	(0%)	1	(0.4%)
Total (synonymous)	160		201		257		159		175		827		230		168		258	

#### Validation cohort

The validation cohort was collected from the archive of the Department of Pathology, University Hospital Schleswig-Holstein, Campus Kiel. The cohort included 463 patients who had undergone either a total or partial gastrectomy for adenocarcinoma of the stomach or esophagogastric junction between 1997 and 2009 and met all inclusion and none of the exclusion criteria. All tissue samples originated from routine therapeutic surgeries, for all of which the patients had given written informed consent. The following patient characteristics were retrieved: type of surgery, age at diagnosis, gender, tumor size, tumor localization, tumor type, depth of invasion, number of lymph nodes resected and number of lymph nodes with metastases. Patients were included if an adenocarcinoma of the stomach or esophagogastric junction was histologically confirmed. Exclusion criteria were defined as (1) histology identified a tumor type other than adenocarcinoma, and (2) patients had undergone perioperative or neoadjuvant chemo- or radiotherapy. Each resected specimen had undergone gross sectioning and histological examination by trained and board-certified surgical pathologists. For outcome analyses, the dates and causes of patients’ deaths were obtained from the Epidemiological Cancer Registry of the state of Schleswig-Holstein, Germany, thereby distinguishing between tumor-related deaths and deaths from other causes. Follow-up data of those patients who were still alive were retrieved from hospital records and general practitioners. All patient data were pseudonymized after study inclusion.

### DNA sequence analysis (discovery cohort)

Genomic DNA was extracted from frozen tissue using the QIAamp DNA mini kit (Qiagen, Hilden, Germany). Cryosections were prepared prior to DNA isolation to guarantee tumor cell content. DNA Exome libraries were prepared using the Nextera Rapid Capture Enrichment Kit, CEX version (Coding Exome Oligos; Illumina, San Diego, USA). Sequencing was performed on a Hiseq4000 instrument (Illumina) with 1% phiX (v3, Illumina) spike-in at 2*75 bp paired-end settings with the 150 bp SBS chemistry. The purity- and ploidy status of the discovery cohort (per case) are shown in Additional file 2: Table S1. Sequencing statistics are summarized in Additional file 2: Table S2.

### DNA isolation from formalin-fixed and paraffin-embedded tissue specimens

Genomic DNA was extracted from formalin-fixed and paraffin-embedded tissue using the QIAamp DNA mini kit (Qiagen, Hilden, Germany). Tissue sections were manually microdissected prior to DNA isolation to ensure a tumor cell content of higher than 80%. The integrity and amplifiability of the isolated DNA was evaluated by a qualitative size-range PCR assay.

### Primary data analysis

Raw fastq data were quality-trimmed, and adapter sequences were removed using bbduk from the BBTools suite version 36.32 (http://sourceforge.net/projects/bbmap) with the following parameters: minlen=25 qtrim=rl trimq=10 ktrim=r k=25 mink=11 hdist=1 overwrite=true tbo=t tpe=t. The Burrows-Wheeler aligner 0.7.15 (https://arxiv.org/abs/1303.3997) with default parameter settings was used to align the sequencing reads to the human reference genome (hs37d5). Duplicate reads were marked with sambamba 0.6.3 [10] and indel realignment was performed using ABRA version 0.97 [11]. FastQC 0.11.5 (http://www.bioinformatics.babraham.ac.uk/projects/fastqc/) and Qualimap 2.2 [12] were used to perform quality checks on the fastq and bam files respectively.

### Somatic mutation calling

Paired tumor-normal variant calling was performed using VarDict 1.5.1 [13] with the following parameters: mapping quality *Q* = 10, base quality phred score *q* = 20, minimum allele frequency *f* = 0.01, and number of nucleotides to extend for each segment *x* = 2000. Additionally, the read position filter *P* = 0.9 and maximum number of reads with mismatches *m* = 4.25 were supplied to var2vcf_paired.pl with further downstream filtering steps to improve detection of low frequency variants as described by Brad Chapman (http://bcb.io/2016/04/04/vardict-filtering/). ANNOVAR [14] was used to annotate variants utilizing refGene, cosmic84, clinvar_20170905, icgc21, nci60, exac03, exac03nontcga, snp142, avsnp150, 1000g2015aug_all, ljb26_all, dbnsfp33a, and intervar_20180118 databases. Homopolymer regions were marked using vcfpolyx from the jvarkit suite (https://github.com/lindenb/jvarkit). Variants were retained if the following criteria were met: allele frequency (AF) ≥ 5%, total read depth ≥ 10 in either the tumor or the normal sample, variant depth in tumor sample ≥ 2, no strand bias, AF > 10% in homopolymer regions. Variants in blacklisted regions described by Fuentes et al. [15] as well as ENCODE [16] were filtered out. In addition, annotation-based filtering was utilized to retain only coding, non-synonymous variants with ExAC AF < 0.01 [17]. Variants unknown to either COSMIC or ICGC were retained only if they occurred within one of the 719 genes of the COSMIC Cancer Gene Census (https://cancer.sanger.ac.uk/census, downloaded June 12, 2018) [18] (https://dcc.icgc.org/). An additional variant recovery process on the list of filtered variants was implemented on a per-patient basis to recover variants from individual samples with AF < 5% if they were present in at least one other sample of the same patient with AF ≥ 5%. Tumor purity and ploidy were computationally estimated using Sequenza [19]. Clonality and cancer cell fraction (CCF) for each variant was determined using Palimpsest [20]. In brief, CCF is computed by adjusting the variant allele fraction for the tumor purity and the absolute copy number at each locus in tumor and normal cells. Mutations were classified as subclonal if the upper boundary of the 95% confidence interval was below the threshold of 0.95. COSMIC Mutational Signatures Version 2 were inferred using the R package deconstructSigs [21].

### Copy number profiling

Allele-specific copy number calling was done using CNVkit 0.9.5 [22]. A pooled normal reference was created from the nine matched non-neoplastic stomach mucosa samples. The initial segments were called with a conservative significance threshold of *t* = 1e−6 and low coverage segments were dropped. Segments were then used along with raw variant calls from VarDict, the estimated tumor purity, to call major and minor copy number variants and annotated by ANNOVAR based on the refGene database. Calls were considered as deletions when total copy number was 0, and as amplifications when total copy number was at least 6.

### Tumor mutation burden, microsatellite instability, and viral sequence analysis

Tumor mutation burden was calculated for each sample in terms of the number of non-synonymous variants per 1 Mb and scaled according to the exome panel size. Microsatellite instability (MSI) status was determined by MSIsensor [23] with a threshold of < 10% for MSS (microsatellite stable), < 10% and > 30% for MSI-L (low), and > 30% for MSI-H (high). To screen for viral integration events, unmapped reads were aligned against a sequence database of 198 human viruses (EBV, human papilloma virus, herpes simplex virus, among others).

### Phylogenetic analysis

Patient-specific multiregional trees from CCF data were constructed using LICHeE [24]. Since LICHeE is limited to constructing trees based on single-nucleotide variations (SNV) only, copy number variants (CNV) were manually incorporated into the SNV-based trees. Driver CNVs as identified by Cancer Genome Interpreter were added to the SNV-based trees; for some patients, this did not result in any changes to the tree nodes, whereas for some patients the internal tree nodes were redrawn to reflect the additional CNVs. In addition, we constructed maximum parsimony trees based on a binary matrix of SNVs per patient using a branch-and-bound algorithm with PHYLIP (Felsenstein, J. 2005. PHYLIP (Phylogeny Inference Package) version 3.6. *Distributed by the author; Department of Genome Sciences, University of Washington, Seattle*).

### Assessment of neutrality

Variant allele frequency (VAF) histograms were used to test the neutral model of cancer evolution as described by Williams et al. [25] using their neutralitytestr package.

To reduce the probability that apparent deviation from neutrality is caused by increase in allelic frequency due to gene duplication events, all variant alleles that had likely undergone gene doubling were removed, as shown by Williams et al. [25]. All detected non-synonymous and synonymous mutations were included since a larger number of passenger mutations whose frequency had been increased by advantageous mutation supports the detection signs of selection [26].

We conducted our analysis on VAFs without the purity correction, assuming that sample purity affects all variant frequencies equally. Therefore, a correction is unlikely to increase the resolution of our analysis. In addition, spatial constraints can introduce sampling bias into patterns of the clonal selection of the tumor [27]; therefore, the analyses also included the average frequency of mutations from all available samples.

### Validation analyses using Sanger sequencing, pyrosequencing, and digital polymerase chain reaction

In order to validate the heterogeneous mutational patterns, Sanger sequencing analysis of *ASXL3*, *TP53* (exon 5 and 8), and *SMAD4* (exon 2, 9 and 11) was done using the PyroMark PCR Kit (Qiagen). PCR products were purified using the NucleoSpin® Gel and PCR Clean-up (Machery-Nagel, Düren, Germany) and sequenced by dye terminator cycle sequencing (BigDye Terminator v1.1 Cycle Sequencing kit, Applied Biosystems, Darmstadt, Germany) with universal M13- or PCR Primers. The sequencing products were purified using the DyeEx 96 Kit (Qiagen) and analyzed on a Genetic Analyzer 3500 (Applied Biosystems). Pyrosequencing, using the PyroMark PCR Kit (Qiagen) and the PyroMark Gold Q24 Reagents (Qiagen), was done to detect SNPs in *BRCA1*, *BRCA2*, *CDH1*, *CTNNB1*, *KRAS*, *MLH1*, *MUTYH*, *PIK3CA*, *POLE*, and *RNF43*. The PyroMark Q24 System and PyroMark analysis software (both Qiagen) were used for analysis. To validate low frequent mutations in *ARID1A*, *ARID1B*, *AKT1*, *CLOCK*, *FLT4*, *IKBKB*, *IKZF3*, *LRP1B*, *MAP2K4*, *MCM8*, *PAX5*, *PRRC2A*, and *TP53BP* digital PCR were done using the ddPCR™ Supermix for Probes (No dUTP) and the QX200™ Droplet Digital™ PCR System (both Biorad) following the manufacturer’s instructions. The primer sequences used are listed in Additional file 2: Table S3. Additional file 2: Table S4 summarizes the validated mutations.

### Histology

Tissue specimens used for histology and immunohistochemistry were fixed in formalin and embedded in paraffin. Deparaffinized sections were stained with hematoxylin and eosin. Histological examination of primary tissue sections was carried out for all cases (discovery and validation cohort) to assure if inclusion criteria were met. Tumors were classified according to the Laurén classification [28]. pTNM stage of all study patients was determined according to the eighth edition of the UICC guidelines [29].

### Immunohistochemistry and scoring of SMAD4 and p53 immunostaining

Immunohistochemistry was carried out with antibodies directed against SMAD4 (dilution 1:50; monoclonal rabbit; 50, Cell Signaling Technology Europe, Frankfurt am Main, Germany), PD-L1 (dilution 1:100, E1L3N, Cell Signaling Technology), and p53 (dilution 1:100, clone DO-7, Novocastra, Leica Microsystems GmbH, Wetzlar, Germany), using whole tissue sections. Immunostaining was performed with the autostainer Bond™ Max System (Leica Microsystems GmbH, Wetzlar, Germany). The immunoreaction was visualized with the Bond™ Polymer Refine Detection Kit (brown labelling; Novocastra; Leica Microsystems, Wetzlar, Germany).

Scoring of each tumor for SMAD4 and p53 expression was assessed by determining a histoscore (H-score), following a semi-quantitative approach combining both the immunostaining intensities (subsequently referred to as IHC scores) and the percentages of positive cells of the tumor. The IHC score was based on tumor cells showing either strong (3+), intermediate (2+), or weak (1+) staining of SMAD4 in the cytoplasm and nucleus, respectively, or of p53 in the nucleus. Tumor cells without detectable cytoplasmic or nuclear staining were scored with 0. The percentage of positive tumor cells (approximated to the nearest 10) showing the defined staining intensities (3+, 2+, 1+, 0) was gauged with respect to all tumor cells visible on each tissue specimen and always added up to a total of 100% tumor cells. Finally, a H-score was calculated according to the formula: H-score = [0 × percentage of immunonegative tumor cells] + [1 × percentage of weakly stained tumor cells] + [2 × percentage of intermediately stained tumor cells] + [3 × percentage of strongly stained tumor cells]. The maximum possible H-score was 300, if all cells of a given tumor sample showed a strong staining: [0 × 0%] + [1 × 0%] + [2 × 0%] + [3 × 100%] = 300. The multipliers within the formula yielded an improved stratification of the H-scores: tumor samples with a predominantly high staining intensity and such samples with a predominantly low staining intensity were more distinctively separated.

The H-score of p53 was divided into four quartiles as described previously [30]. The outer (Q1: H-score ≤ 15 and Q4: H-score ≥ 189) and inner quartiles (Q2: H-score = 16–91 and Q3: H-score = 92–188) were joined to form two new groups: “Q1/Q4” and “Q2/Q3”. Here, the “Q1/Q4” group was assumed to indicate cases with mutated *TP53* [30].

### MDM2 fluorescence in situ hybridization

Analysis of MDM2 amplification was done by fluorescence in situ hybridization using the Vysis MDM2/CEP 12 FISH Probe Kit (Abbott Diagnostika MediSense, Wiesbaden, Germany) following standard procedures. The results of FISH were evaluated by screening the entire tissue sections. Subsequently, MDM2 and centromer 12 signals were counted in at least 20 representative adjacent cancer cell nuclei within the invasive region. The presence of FISH clusters was noted and the ratio of *MDM2*/centromer 12 signals was calculated. The gene count was calculated by dividing the number of *MDM2* gene signals by the number of cancer cell nuclei studied.

### Assessment of further clinicopathological characteristics

*H. pylori* [31], Epstein-Barr virus [4], microsatellite (MSI) [6], HER2 status [9], and *TP53* genotype [30] were assessed as described previously.

### Statistical methods

SPSS version 24.0 (IBM Corp., Armonk, NY, USA) was used for statistical analyses. The correlation between non-ordinal clinicopathological patient characteristics and SMAD4 was tested with Fisher’s exact test. T category, N category, UICC stage, and tumor grading as variables of ordinal scale were tested with Kendall’s tau-test. Median survival with 95% confidence intervals was determined by the Kaplan-Meier method. Differences between median survivals were tested with the log-rank test. A multivariate survival analysis (Cox regression) was performed. A *p* value of ≤ 0.05 was considered to be significant. All *p* values are given uncorrected. The Siemes (Benjamini-Hochberg) procedure was applied to compensate for false discovery rate. Any *P* values that lost significance are marked.

## Results

### Patient cohort for whole-exome sequencing (discovery cohort)

**Fig. 1:**
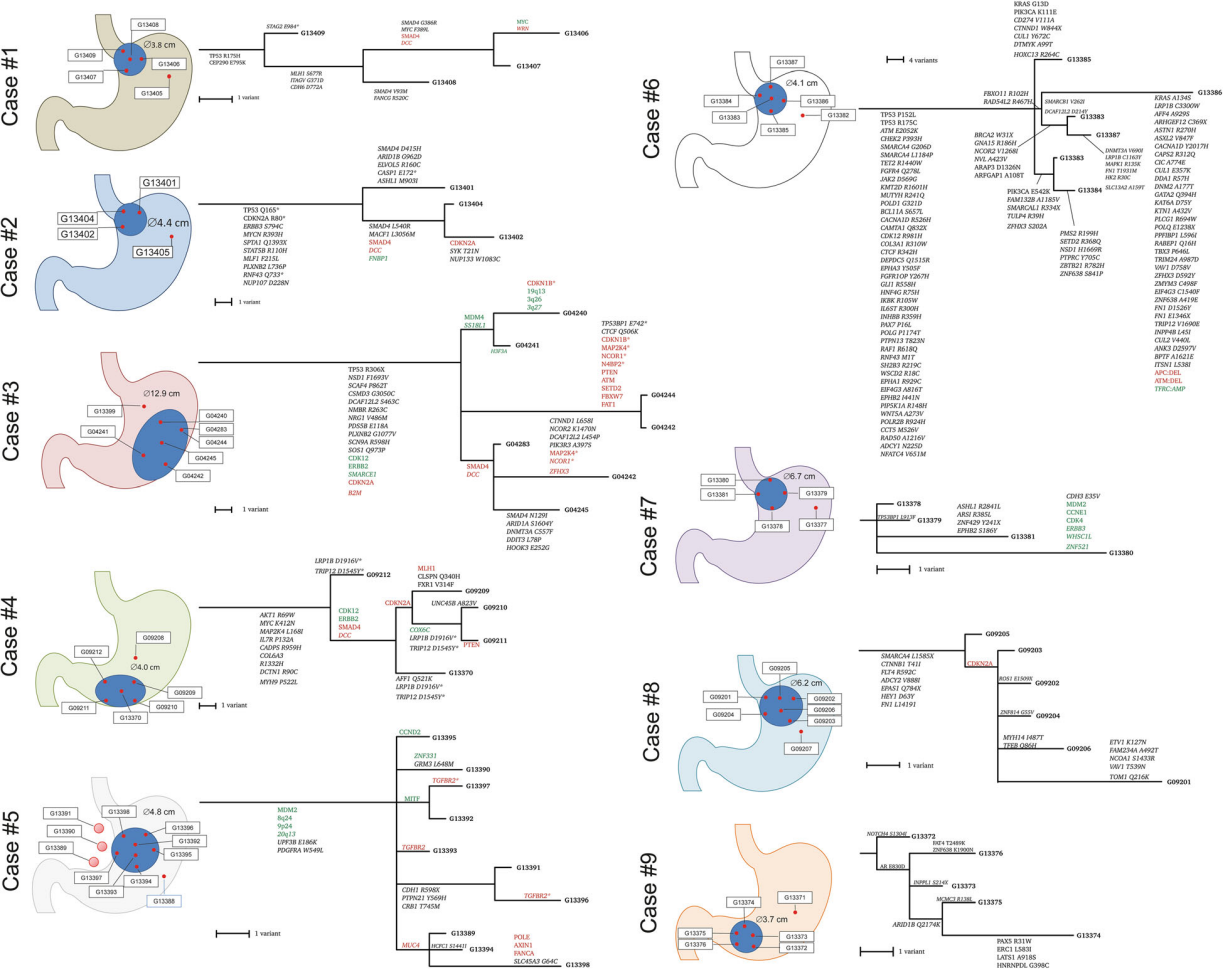
Discovery cohort and multiregional trees. Schematic representation of the nine patients from the discovery cohort. Multiregional trees provide evidence of somatic evolution. Text in green and red indicate amplifications and deletions; italics represent predicted drivers, while others are known drivers as determined by Cancer Genome Interpreter. Variants denoted by an asterisk are those that are present in more than one branch of a tree and could not be satisfactorily resolved into a single branch

The clinicopathological characteristics of the discovery cohort are summarized in Table 1. A total of 45 samples were obtained from the primary tumors. In a single case, three samples were collected from three separate lymph node metastases (in total 3 to 10 tumor samples per case; Table 1; Fig. 1). Including the non-neoplastic stomach mucosa, we finally obtained whole-exome sequences from 57 tissue samples (4 to 11 samples per case). For further details on sequencing data, see Supplemental Results (Additional file 3: Results R1) [32, 33].

### Gastric cancer shows substantial genetic variability within its lateral expansion and between primary tumor and lymph node metastases

**Fig. 2:**
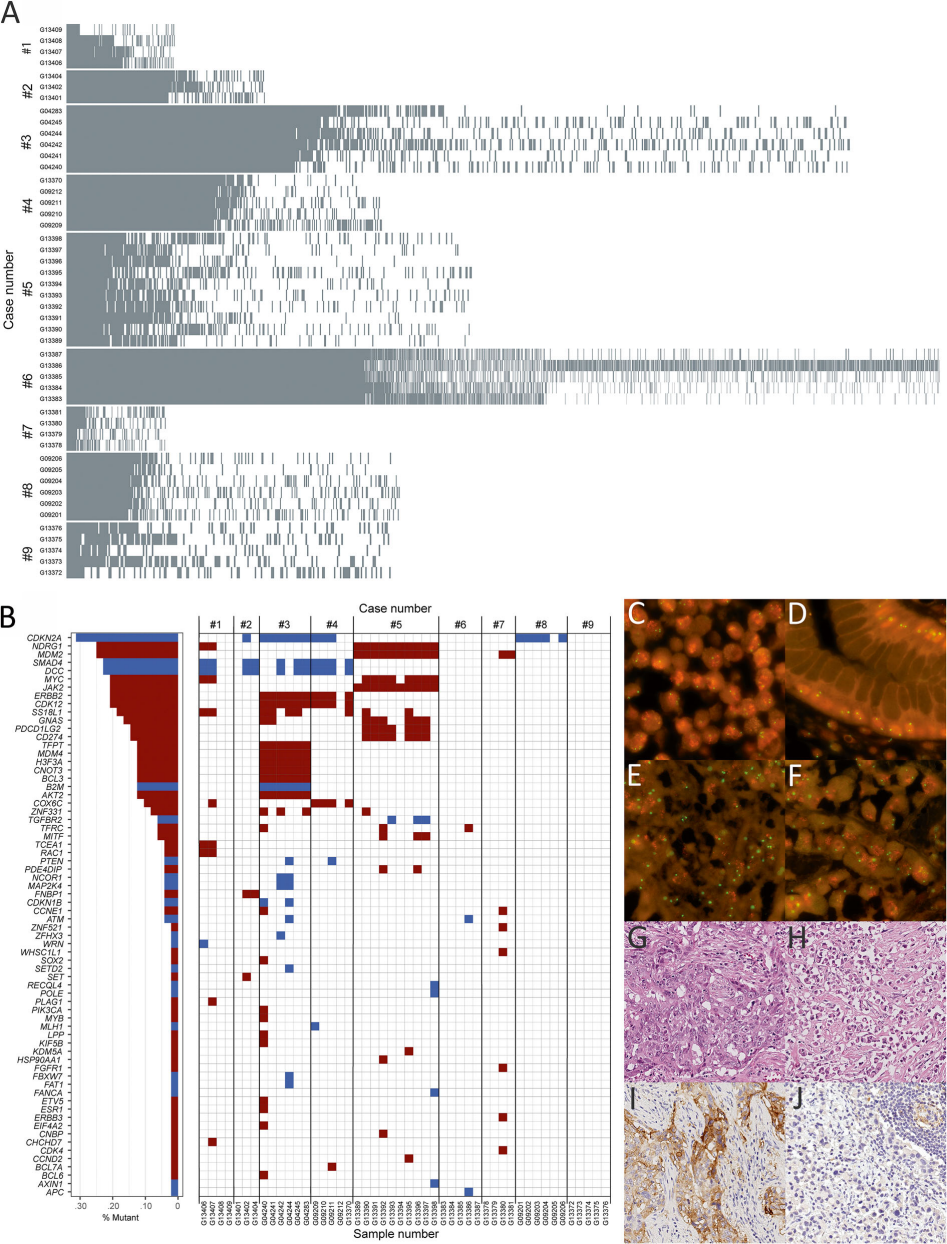
Intratumoral heterogeneity and Copy number variation. **A** Non-synonymous mutations were unevenly distributed among patients and patient samples. Each row represents a patient sample and each column represents one non-synonymous mutation. **B** Copy number variation analyses showed marked intratumoral heterogeneity (maroon denotes amplification and dark blue deletion). **C–J** Case #5 yields homogeneous amplifications in *MDM2* (all ten samples) and a heterogeneous amplification of *CD274* (PD-L1; 2/10 samples including a single lymph node metastasis). *MDM2* amplification was confirmed independently in all samples, i.e., primary tumor (**C, F**; non-neoplastic mucosa as a reference in **D**) and all lymph node metastases (**E**). Amplification of *CD274* was associated with strong PD-L1 immunostaining only in a single sample (**I**) and only in a single lymph node metastasis. All other samples were immunonegative for PD-L1 (**J**). The PD-L1-positive tumor area (**G**) showed a phenotype, different from the remainder (**H**). Primary tumor (**C**); corresponding non-neoplastic mucosa (**D**); lymph node metastasis corresponding to sample G13390 (**E**, **G**, **I**) and a sample of the primary tumor without *CD274* amplification (PD-L1-immunonegative; **F**, **H**, **J**). Fluorescence in situ hybridization (orange signal: MDM2, green signal: reference centromere; **C**–**F**); H&E staining (**G**; **H**) and anti-PD-L1-immunostaining (**I, J**). Original magnifications 1000-fold (**C**–**F**), 400-fold (**G–J**)

In a reductionist view, GC may serve as two-dimensional model of lateral cancer expansion, i.e., along the planes of the stomach wall, shedding some light on the evolutionary biology of GC, and helping to unravel obstacles of precision medicine. First, we explored intratumoral heterogeneity of GC specimens obtained from different sites of the lateral expansion of the primary tumors and from different lymph node metastases. The discovery cohort harbored 16,537 non-synonymous mutations (i.e., missense, nonsense and frameshift; Additional file 2: Table S5-S6). In the individual patient, the number of non-synonymous mutations ranged from 181 to 3111 (median: 369), and in the individual tumor sample, it ranged from 49 to 2348 (median: 159). The highest mutational burden was found in the MSI GC (Table 1; Fig. 2A). Between 8.7 and 46.8% of the non-synonymous mutations were found in all samples of the same patient (Table 1). The vast majority of non-synonymous mutations (53.2-91.3%) was not present in each sample of the individual patient. With regard to all non-synonymous mutations (*n* = 4413 genes), the vast majority, i.e., 3272 (74.1%) genes, were not found in every sample (Table 1).

In a single case, additional sequence data were obtained from three separate lymph node metastases. Similarly, the number and type of non-synonymous mutations varied between each lymph node metastasis and between the primary tumor and the three lymph node metastases. Interestingly, 172 mutations present in the primary were not detected in the lymph node metastases, while 58 mutations found in the lymph node metastases were absent in the samples obtained from the primary tumor (Additional file 2: Table S7). Collectively, these data show that the lateral expansion of GC is associated with substantial genetic variability, and lymph node metastases may stem synchronously from different areas of the primary tumor. Thus, the analysis of a single sample, including lymph node metastases, may miss between 53.2 and 91.3% of the mutations present in GC.

### Intratumoral heterogeneity also applies to copy number variations

Previously, it has been shown that intratumoral heterogeneity also applies to copy number variations (CNV) and we next assessed the extent of intratumoral heterogeneity along the lateral expansion for CNVs [7, 9, 34]. A total of 219 genes showed CNVs. The number of genes with CNVs per case ranged from 0 to 98 (Additional file 2: Table S8; Fig. 2B). The highest number was found in case #5. Interestingly, this case did not have any obvious drivers in terms of SNVs and showed strong amplifications in *MDM2*, *CD274* (PD-L1), *JAK2*, *MYC*, and *NOTCH2* as well as deletions in *POLE1* and *TGFBR2* in individual samples. Across all samples, we found examples for *HER2* (validated independently by in situ hybridization; case #3 and #4), *MYC* (case #5), and *CDK12* (case #3 and #4) amplifications as well as *CDKN2A* (case #2, #3, #4 and #8), *TP53* (case #3), and *PTEN* (Case #3 and #4) losses (Additional file 2: Table S8). The homogeneous amplification of *MDM2* and the heterogeneous amplification of *CD274* (PD-L1) in case #5 were validated independently by in situ hybridization (MDM2) and immunohistochemistry (PD-L1; Fig. 2C–J).

Only in three cases (#3, #4 and #5), a total of 16 genes showed CNVs in all samples of the individual patient. The vast majority of CNVs was not present in every sample and single-sample analysis may miss between 71.4 and 100% of the CNVs present in a tumor. These data show that genetic heterogeneity of CNVs along the lateral expansion is substantial and does not apply only for genes of tyrosine kinase receptors but also for other putative druggable targets such as PD-L1 (Additional file 2: Table S8; Fig. 2G–J).

### Genetic variability along the lateral expansion compromises the discovery of clonal mutations

The analysis of a single tissue specimen carries a risk of mis-interpreting subclonal mutations as clonal (“clonal illusion”) or to miss important mutations, which are relevant for disease progression and therapy response [35]. In view of the marked intratumoral heterogeneity along the lateral expansion present in our discovery cohort, we then assessed the risk of clonal illusion. We analyzed the cancer cell fraction (CCF) on a per-sample basis and on a per-patient basis (Table 1; Additional file 2: Table S9). On a per-sample basis, the percentage of clonal SNVs ranged from 1 to 99% for non-synonymous mutations (Additional file 2: Table S5) and from 0 to 98.6% for synonymous mutations (Additional file 2: Table S6). However, when clonality was assessed on a per-patient basis (all data for each patient were combined into a “single sample”), the number of clonal mutations ranged from 0 to 80 (median 1.0) for non-synonymous mutations and from 0 to 32 (median 1.0) for synonymous mutations (Table 1). However, the *TP53* mutations in case #1, #2, and #3 were classified as clonal in the per-sample *and* in the per-patient analysis. These data show that a single-sample analysis cannot reliably assess the true clonality status of a somatic mutation and that the vast majority of the mutational landscape of GC is subclonal.

**Fig. 3:**
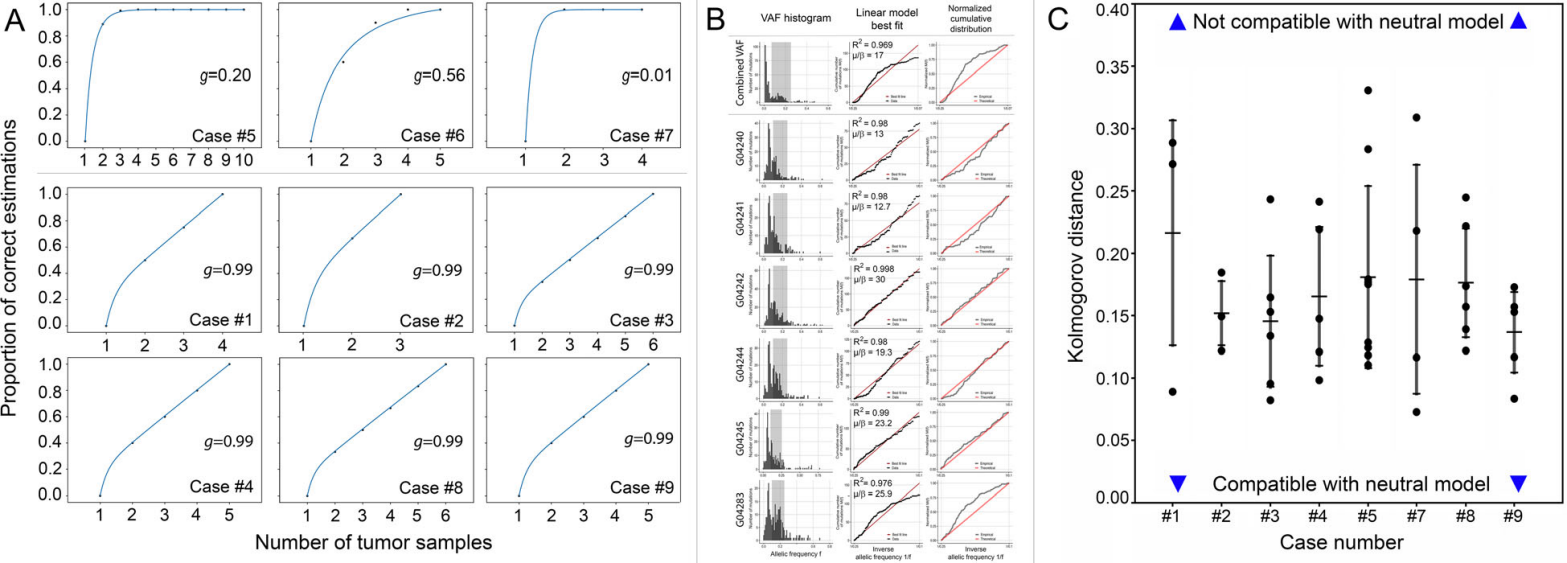
Clonality and neutrality in the discovery cohort. **A** Clonality was assessed as described [36, 38]. Cases #1, #2, #3, #4, #8, and #9 are highly unbalanced and additional samples would be needed for correct estimation of clonality. In three cases (cases #5, #6, and #7), we could be fairly certain that mutations from the root of the phylogenetic tree were indeed clonal using the existing number of samples. **B, C** The neutral model assumes that there are no selective differences, such that the number of mutations of a certain allelic frequency declines as the inverse of that frequency [38]. Here, we show the agreement between each tumor sample and this neutral expectation. **B** Illustrates neutrality analysis of the samples from case #3. Left column: variant allele frequency histogram. Dark gray shade marks interval used for comparison with the neutral model. Central column: shows increment in the cumulative number of mutation with inverse allelic frequency 1/f (black dots) and linear model best fit (red line). Light gray marks samples that are in agreement with the neutral model *R*2 ≥ 0.98. Right column: normalized cumulative distribution of mutations and theoretical model. Distance between distributions was quantified using a Kolmogorov-Smirnov test. While the figure for the combined VAF shows deviations from neutrality, here mostly driven by sample G04283, some parts of the tumor could still evolve under neutral conditions. **C** Summarizes neutrality analyses for cases #1 to #5, #7 to #9. Case #6 (MSI) was not included in the neutrality analysis as a large, likely clonal, peak covered the most of the frequency range obfuscating the distribution of subclonal mutations. The agreement is quantified by the Kolmogorov-Smirnov test, where the Kolmogorov distance between the empirical and the theoretical distribution is shown for each sample. The normalized cumulative number of putatively subclonal mutations in a frequency area below the clonal peak was used where a power-law distributed subclonal tail of mutations would be expected in the model of neutral evolution. The lines represent the standard deviation of the Kolmogorov distance across samples per patient

To further assess the reliability of the clonality assessment, we estimated the number of samples required for correct identification of clonal mutations by using the approach described by Opasic et al. [36] and Werner et al. [37]. First, the “balance factor” *g* was assessed for every individual tumor by fitting the information gain with each multiregion sample to a theoretical curve. Next, the information from the fully reconstructed multiregional trees and the branch-defining subclonal alterations were used (Fig. 3A). Six (case #1, #2, #3, #4, #8, #9) tumors were considered to be highly unbalanced, which implies that the number of “truly” clonal mutations was indeed low. A reliable identification of these mutations would require the sequencing of many additional tumor samples. In one patient (case #6) with a fairly balanced phylogenetic tree (*g* = 0.56), five samples were sufficient for the identification of truly clonal mutations with a probability > 90%. For two other patients (case #5 and #7) with low estimated values of *g* (*g* = 0.2 and *g* = 0.01) we could be fairly certain (> 98% probability) that mutations from the root of the multiregional tree were indeed clonal using the existing number of samples (Fig. 1). No difference was found between patients receiving perioperative treatment and therapy-naïve patients (data not shown). Collectively, these data show that the assessment of clonality depends on the extent of interindividual variability of intratumoral heterogeneity along the lateral expansion, hence the number of samples studied, and that even multiregional sequencing carries a risk of mis-interpreting clonality (Table 1).

### Different modes of cancer evolution might be operating in GC

Cancer is an evolutionary disease and the main driver of intratumoral heterogeneity [39, 40]. It is conceptually similar to the evolution of asexual microorganisms and four models of evolution are discussed [41]: (1) in the “sequential” or “linear” model mutations are acquired linearly in a step-wise process with selective sweeps occurring after driver mutations have been acquired; (2) the “branched” evolution corresponds to a scenario where multiple clones with increased fitness and new driver mutations diverge from a common ancestor and evolve in parallel; (3) “neutral” evolution is an extreme case of “branching” evolution in which there is no selection of fitness changes during the lifetime of the tumor. In the neutral model, cancers acquire all tumor-driving alterations responsible for cancer expansion in the first malignant cell. Thereafter, the cancer expands and neutral variation is generated, reflected by a large number of (probably non-functional) passenger mutations that are responsible for the extensive and common intratumoral heterogeneity also found in our discovery cohort; finally, (4) “punctuated” evolution or the “Big Bang” model of evolution refers to a model where a large number of genomic alterations occur in short bursts of time, at the earliest stages of tumor progression (“Big Bang”) with heterogeneity being high at tumor initiation and one/few dominant clones expand to form the tumor mass.

There is emerging evidence that modes of evolution may undergo transitions over time, or that multiple modes may be operating concurrently for different classes of mutations [41]. In the next set of data analyses, we explored the risk of mis-interpreting the mode of evolution by single-sample analysis. We compared the variant allele frequency (VAF) histograms with the neutral model of cancer evolution as described initially by Williams et al. [38] (Fig. 3B; Additional file 1: Figure S2).

The VAF histograms (Fig. 3B; Additional file 1: Figure S2) show that the majority of VAF distributions found in individual samples is compatible with a neutral expectation, i.e., no selection. However, there is a high degree of heterogeneity in deviation from neutrality within each tumor, and VAF profiles collected from individual samples can lead to very different results for the evolutionary dynamics of cancer compared to the combination of all samples (Fig. 3C). These findings support the contention that different modes of evolution (e.g., neutral vs. non-neutral) might be operating concurrently in different parts of the same GC.

### Multi-sample tree-based analyses

The temporal phylogenetic order and spatial distribution of mutations not only provides insights into cancer evolution, but also might provide evidence of parallel evolution and epistatic interactions and infer clues about their tumor biological relevance, e.g., for tumor progression. Therefore, we next generated multiregional trees based on driver SNVs and CNVs. As shown in Fig. 1, all tumors of our cohort provided evidence of branched somatic evolution, with the most complex being the MSI GC, confirming data published recently by von Loga et al. [8]. In addition, we inferred maximum parsimony trees in accordance with Lee et al. (Additional file 1: Figure S3) [5]. The authors observed a common phylogeny pattern of five cases with GC in which the primary genome is branched from a trunk while all the lymph node genomes (*n* = 3 for each of the 5 cases) cluster in a separate branch. We did not observe this pattern for the single sample (case #5) for which lymph node data was available. In contrast to Lee et al. [5], in case #5 the lymph node metastases did not cluster together in a separate, distinct branch, but rather clustered with different individual samples from the primary tumor (Fig. 1; Additional file 2: Table S7). This further substantiates that lymph node metastases may stem from different areas of the primary tumor, “synchronously.” Comparing the multiregional trees with the maximum parsimony trees following the Lee et al. [5] methodology, one can observe similar trees with differences attributed to methodology as well as the additional CNV data included in the multiregional trees. Generating LICHeE-based multiregional trees including passenger mutation and/or synonymous mutations generated similar relationships in the trees (data not shown).

It was interesting to note that the *SMAD4* mutations of four cases with *TP53* mutations (i.e., cases #1, #2, #3, and #4) were subclonal and that different mutations of *SMAD4* aligned with different subclones (Fig. 1).

### Multiregional sequencing and pathway analysis provide evidence of parallel evolution

Since tumor progression and spatial separation of tumor subclones support parallel evolution, i.e., different subclones evolving in parallel acquire distinct mutations in the same gene (e.g., *SMAD4*) and/or pathway (e.g., TGFβ-pathway), we next sought our data set for further evidence of parallel evolution: 369 genes of our discovery cohort harbored two to four different non-synonymous mutations in the same patient (Additional file 2: Table S10), some of which could be due to parallel evolution. We next assigned non-synonymous mutations and CNVs to pathways, which have been linked to GC biology, i.e., the SWI/SNF, TGFβ, Hippo, sonic hedgehog, NOTCH, WNT and JAK-STAT (Additional file 2: Table S11), and generated multi-sample trees. The listed pathways had been chosen based on domain expertise as driver aberrations tend to occur less frequently in multiple genes within a given pathway thereby compromising pathway enrichment analyses.

**Fig. 4:**
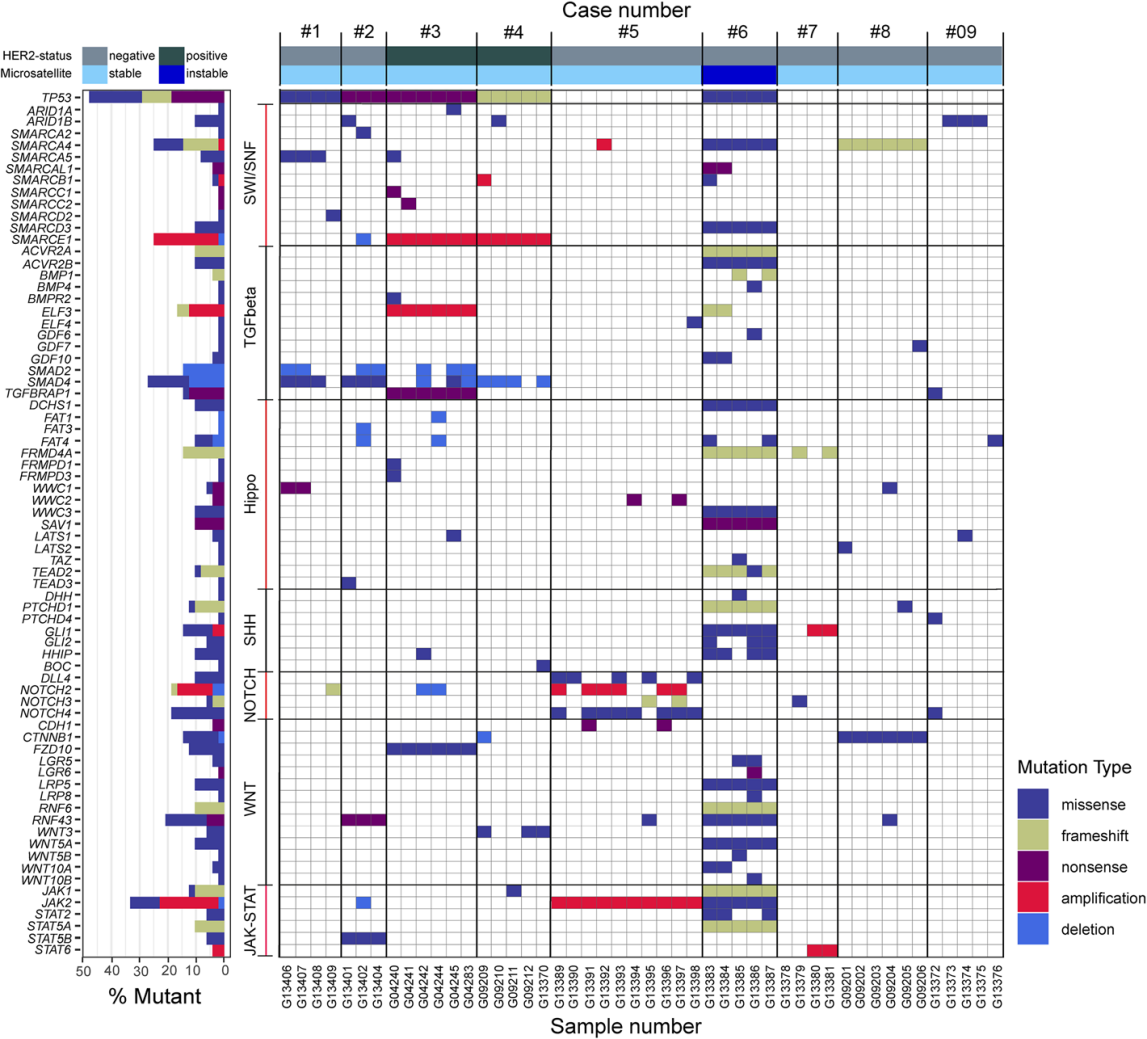
Pathway analysis. Assignment of mutations to pathways, i.e., the SWI/SNF, TGFβ, Hippo, sonic hedgehog, NOTCH, WNT, and JAK-STAT pathway, also showed marked intratumoral heterogeneity and provided evidence of parallel evolution

The MSI GC showed the highest number of mutations (*n* = 10) in a single pathway, while the remainder showed alterations in 1–5 genes per pathway (Fig. 4; Additional file 2: Table S11). Interestingly, while excluding the MSI GC, we noted pathway-related clustering of mutations in individual cases, e.g., the SWI/SNF (case #1, #2, #3, #4), TGFβ (case #1, #2, #3, #4), and NOTCH pathway (case #5) (Fig. 4; Additional file 2: Table S11).

Recently, Park et al. [42] demonstrated in animal models that SMAD4 cooperates with p53 loss to promote the development and metastatic progression of GC. In support of the findings, our pathway analysis shows that four of five cases with *TP53* mutations (including all three cases with clonal *TP53* mutation) also had alterations in the TGFβ-pathway. Furthermore, *SMAD4* mutations and losses were only found in *TP53* mutant cases (Additional file 2: Table S11). In addition, all *TP53* mutant cases showed alterations in the SWI/SNF pathway validating and extending findings made by Frankell et al. [43]. He et al. [44] provided evidence that members of the SWI/SNF pathway regulate cellular senescence via the p53/p21 and p16/pRB pathways. Collectively, these findings support the contention that parallel evolution is operative in GC and may affect the same gene or occur within different genes of the same pathway. It also points towards deterministic trajectories, where a specific ordering of mutations is advantageous for the tumor [35]: cancer progression in *TP53* mutant GCs depends on subsequent alterations in the TGFβ and SWI/SNF pathway.

### Decreased or lost expression of SMAD4 is associated with an overall worse patient outcome and correlates significantly with p53 expression

**Fig. 5:**
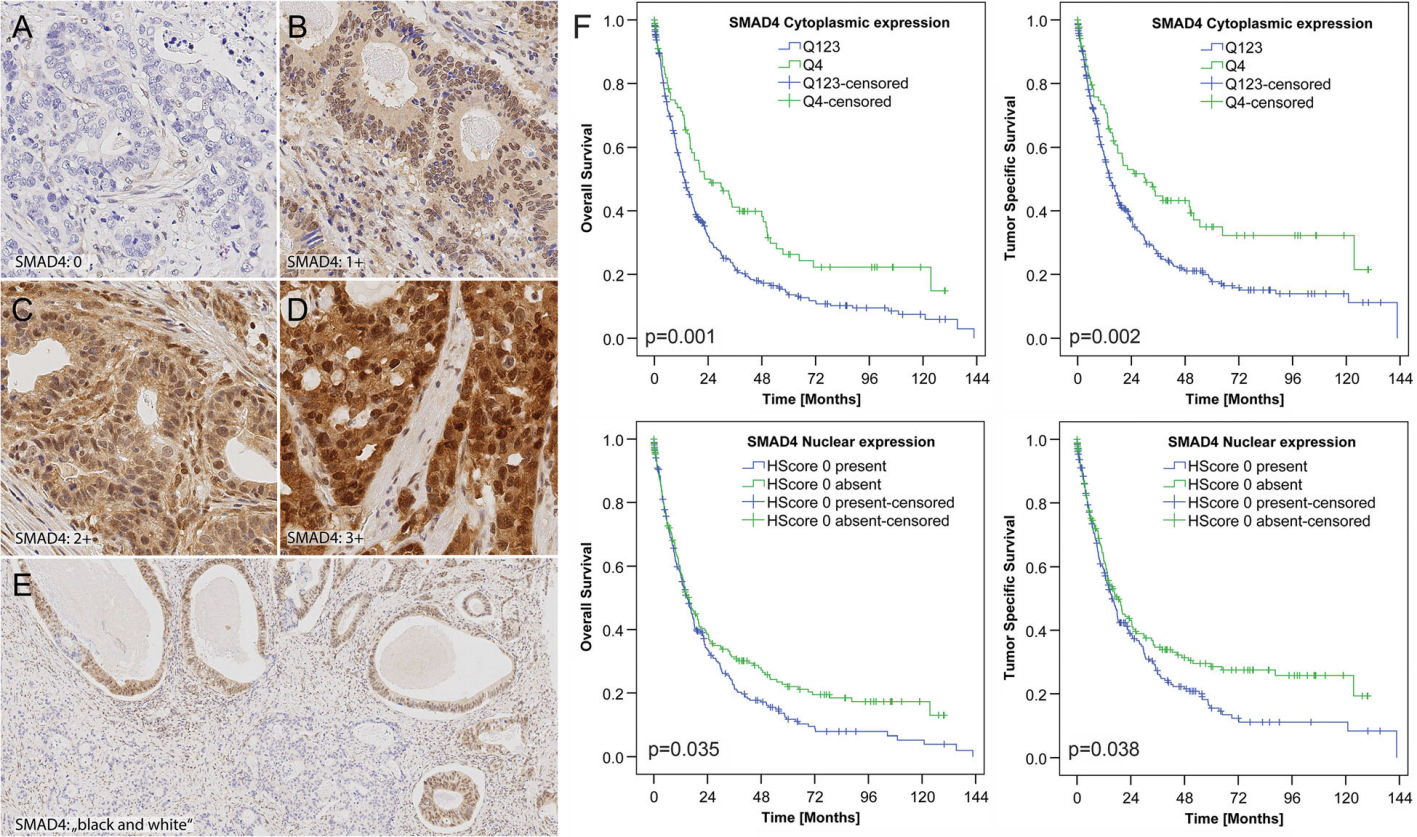
SMAD4 is heterogeneously expressed in gastric cancer and a decreased expression correlates with patient survival (validation cohort). References for immunostaining analysis according to H-score. Staining intensities ranged from 0 (**A**; nuclear and cytoplasmic negative) to 3+ (**D**, nuclear and cytoplasmic strong expression) with 1+ (**B**; nuclear and cytoplasmic weak expression) and 2+ (**C**; nuclear and cytoplasmic moderate expression) in between. Black-and-white expression of SMAD4 describes tumors with clearly demarcated areas of complete loss of nuclear and cytoplasmic SMAD4 expression next to areas with retained expression (**E**). Anti-SMAD4 immunostaining, hematoxylin counterstain; × 400 (**A–D**) and × 100 (**E**) magnifications. **F** Kaplan-Meier curves depicting patients’ survival according to SMAD4 status (Q1–3 vs. Q4; for further details see Suppl. Results). Kaplan-Meier curves demonstrating correlations between cytoplasmic SMAD4 (*top row*) and nuclear (*bottom row*) loss in tumor cells and overall as well as tumor-specific survival

However, if deterministic trajectories are operative and lead to parallel evolution in spatially separated areas of the tumor, this might not inevitably be of biological relevance: epistatic interaction and parallel evolution could be neutral, at least partially. To test this, we next explored the putative significance of SMAD4 alterations on patient prognosis. Using a validation cohort, we aimed to test the hypothesis that a decreased or lost expression of SMAD4 would correlate with clinicopathological patient characteristics in a Caucasian study population. SMAD4 and p53 expression were studied using whole tissue sections and a validation cohort of 463 GCs (Fig. 5; Table 2; for further details see Additional file 3: Results S1).

**Table 2:** SMAD4 expression in the validation cohort and correlation with clinicopathological patient characteristics (*insignificant after correction for multiple testing)

				SMAD4 cytoplasmatic expression					SMAD4 nuclear expression				
		Total valid		Q123		Q4			HScore 0 present		HScore 0 absent		
		***n***	(%)	***n***	(%)	***n***	(%)	***p*** value	***n***	(%)	***n***	(%)	***p*** value
**Total**	**Total**	463	(100)	366	(79.1)	97	(20.9)		263	(56.8)	200	(43.2)	
**Gender**	Female	178	(38.4)	144	(80.9)	34	(19.1)	0.482	90	(50.6)	88	(49.4)	0.034
	Male	285	(61.6)	222	(77.9)	63	(22.1)		173	(60.7)	112	(39.3)	
**Age group**	< 68 years	232	(50.1)	190	(81.9)	42	(18.1)	0.139	131	(56.5)	101	(43.5)	0.925
	≥ 68 years	231	(49.9)	176	(76.2)	55	(23.8)		132	(57.1)	99	(42.9)	
**Localization**	Proximal stomach	145	(31.0)	123	(84.8)	22	(15.2)	0.049	93	(64.1)	52	(35.9)	0.033*
	Distal stomach	318	(69.0)	243	(76.4)	75	(23.6)		170	(53.5)	148	(46.5)	
**Laurén phenotype**	Intestinal	237	(51.2)	186	(78.5)	51	(21.5)	0.313	149	(62.9)	88	(37.1)	0.026
	Diffuse	147	(31.7)	122	(83.0)	25	(17.0)		73	(49.7)	74	(50.3)	
	Mixed	31	(6.7)	24	(77.4)	7	(22.6)		19	(61.3)	12	(38.7)	
	Unclassifiable	48	(10.4)	34	(70.8)	14	(29.2)		22	(45.8)	26	(54.2)	
**Grading**	G1 / G2	107	(23.1)	82	(76.6)	25	(23.4)	0.499	62	(57.9)	45	(42.1)	0.824
	G3 / G4	356	(76.9)	284	(79.8)	72	(20.2)		201	(56.5)	155	(43.5)	
**pT category**	T1a / T1b	58	(12.5)	38	(65.5)	20	(34.5)	0.011*	32	(55.2)	26	(44.8)	0.923
	T2	53	(11.4)	38	(71.7)	15	(28.3)		28	(52.8)	25	(47.2)	
	T3	182	(39.3)	150	(82.4)	32	(17.6)		108	(59.3)	74	(40.7)	
	T4a / T4b	170	(36.7)	140	(82.4)	30	(17.6)		95	(55.9)	75	(44.1)	
**pN category**	N0	132	(28.5)	99	(75.0)	33	(25.0)	0.151	69	(52.3)	63	(47.7)	0.399
	N1	63	(13.6)	48	(76.2)	15	(23.8)		39	(61.9)	24	(38.1)	
	N2	85	(18.4)	70	(82.4)	15	(17.6)		49	(57.6)	36	(42.4)	
	N3a/b	182	(39.4)	148	(81.3)	34	(18.7)		106	(58.2)	76	(41.8)	
**M category**	M0	376	(81.2)	291	(77.4)	85	(22.6)	0.079	217	(57.7)	159	(42.3)	0.471
	M1	87	(18.8)	75	(86.2)	12	(13.8)		46	(52.9)	41	(47.1)	
**UICC stage**	IA / IB	79	(17.1)	55	(69.6)	24	(30.4)	0.011*	40	(50.6)	39	(49.4)	0.832
	IIA / IIB	99	(21.4)	77	(77.8)	22	(22.2)		59	(59.6)	40	(40.4)	
	IIIA / IIIB / IIIC	197	(42.6)	158	(80.2)	39	(19.8)		118	(59.9)	79	(40.1)	
	IV	87	(18.8)	75	(86.2)	12	(13.8)		46	(52.9)	41	(47.1)	
**Lymph node ratio**	Low (< 0.189)	226	(48.9)	175	(77.4)	51	(22.6)	0.426	126	(55.8)	100	(44.2)	0.639
	High (≥ 0.189)	236	(51.1)	190	(80.5)	46	(19.5)		137	(58.1)	99	(41.9)	
**pL category**	L0	216	(48.8)	167	(77.3)	49	(22.7)	0.645	112	(51.9)	104	(48.1)	0.069
	L1	227	(51.2)	180	(79.3)	47	(20.7)		138	(60.8)	89	(39.2)	
**pV category**	V0	393	(88.9)	308	(78.4)	85	(21.6)	0.856	225	(57.3)	168	(42.7)	0.446
	V1	49	(11.1)	38	(77.6)	11	(22.4)		25	(51.0)	24	(49.0)	
**R status**	R0	400	(87.3)	311	(77.8)	89	(22.3)	0.170	230	(57.5)	170	(42.5)	0.479
	R1 / R2	58	(12.7)	50	(86.2)	8	(13.8)		30	(51.7)	28	(48.3)	
**HER2 status**	Negative	397	(91.9)	320	(80.6)	77	(19.4)	0.657	227	(57.2)	170	(42.8)	> 0.999
	Positive	35	(8.1)	27	(77.1)	8	(22.9)		20	(57.1)	15	(42.9)	
***H. pylori*** **status**	Negative	330	(84.6)	261	(79.1)	69	(20.9)	0.092	189	(57.3)	141	(42.7)	0.207
	Positive	60	(15.4)	41	(68.3)	19	(31.7)		29	(48.3)	31	(51.7)	
**EBV status**	Negative	428	(95.5)	341	(79.7)	87	(20.3)	0.273	242	(56.5)	186	(43.5)	0.821
	Positive	20	(4.5)	14	(70.0)	6	(30.0)		12	(60.0)	8	(40.0)	
**MSI status**	MSS	412	(92.2)	332	(80.6)	80	(19.4)	0.008*	236	(57.3)	176	(42.7)	0.375
	MSI	35	(7.8)	21	(60.0)	14	(40.0)		17	(48.6)	18	(51.4)	
**p53 status**	Quartile 1/Quartile 4	226	(49.8)	186	(82.3)	40	(17.7)	0.163	145	(64.2)	81	(35.8)	0.003
	Quartile 2/Quartile 3	228	(50.2)	175	(76.8)	53	(23.2)		115	(50.4)	113	(49.6)	
**Overall survival [months]**	Total / events / censored	451		347 / 289 / 68		94 / 62 / 32		0.001	257 / 210 / 47		194 / 141 / 53		0.035*
	Median survival			13.4 ± 1.1		22.4 ± 7.0			14.9 ± 1.4		15.6 ± 1.9		
	95% C.I.			11.3–15.5		8.7–36.0			12.2–17.6		12.9–17.1		
**Tumor-specific survival [months]**	Total / events / censored	423		333 / 236 / 97		90 / 51 / 39		0.002	240 / 173 / 67		183 / 114 / 69		0.038*
	Median survival			14.7 ± 1.4		30.3 ± 7.5			15.5 ± 1.6		18.4 ± 2.7		
	95% C.I.			12.0–17.3		15.5–45.0			12.4–18.5		13.1–23.8		

A decreased expression of cytoplasmic SMAD4 (Q1-3 vs. Q4) was associated with advanced local tumor growth (T category), UICC stage, and MSI status (Table 2). A decreased nuclear expression of SMAD4 significantly correlated with p53-status assessed by immunohistochemistry (*p* = 0.003; Table 2).

The entire validation cohort showed a median overall survival (OS) of 15.0 months and a median tumor-specific survival (TSS) of 16.6 months. Patient prognosis significantly depended on the Laurén phenotype, T, N, M, L, V, Pn, and R category, UICC stage, lymph node ratio, and cytoplasmic SMAD4 expression. Patients with cytoplasmic SMAD4 loss showed significantly lower median OS (13.4 months, 95% C.I. 11.3–15.5; *p* = 0.001) and TSS (14.7 months, 95% C.I. 12.0–17.3; *p* = 0.002) compared with retained SMAD4 expression (OS: 22.4 months, 95% C.I. 8.7–36.0; TSS: 30.3 months, 95% C.I. 15.5–45.0) (Fig. 5F). The correlation between nuclear SMAD4 expression and OS (*p* = 0.035) and TSS (*p* = 0.038) lost significance after multiple testing (Fig. 5F). However, on multivariate analyses, loss of cytoplasmic SMAD4 expression (HR 1.378; 95%CI 1.022–1.828; *p* = 0.035) was an independent prognosticator of OS (Additional file 2: Table S12).

The *TP53* genotype (exons 4–10) was available from 105 patients (34 cases with and 71 without a mutation or with a silent mutation; Table 2) [30]. Kaplan-Meier curves showed no differences in OS or TSS for cytoplasmic SMAD4 expression and *TP53* genotype (Additional file 1: Figure S5). Interestingly, patients with *TP53* mutation and any loss of nuclear SMAD4 expression showed a lower median survival compared with *TP53* wildtype patients [median OS 9.9 months (95%C.I. 8.2–11.5 months) vs. 13.2 months (95%C.I. 0.0–28.1 months); median TSS 9.9 months (95%C.I. 6.6–12.9 months) vs. 13.2 months (95%C.I. 7.9–18.6 months)]. However, due to low case numbers, this did not reach statistical significance (Additional file 1: Figure S5).

To the contrary, the expression of p53 had been studied previously in the same validation cohort and was shown not to correlate with local tumor growth, nodal spread, or patient prognosis [30].

## Discussion

The heterogeneity of malignant tumors is a major barrier to drug development and long-term disease control [45]. However, comprehensive data on intraprimary and intermetastatic genetic heterogeneity in GC are scarce. Lee et al. [5] performed whole-exome sequencing of 15 pairs of primary GC and their matched lymph node metastases in an Asian patient population and noted a substantial variation in the extent of mutational overlap or mutational heterogeneity between primary and lymph node metastasis genomes. However, Lee et al. [5] studied only a single specimen from the primary tumor and did not explore the risk of sampling error in the primary tumor nor heterogeneity in the lateral expansion. Pectasides et al. [7] analyzed patterns of heterogeneity in two independent patient cohorts. In the first cohort, again only a single biopsy sample was obtained from the primary tumor of 11 patients and was compared with synchronous metastatic biopsies. In a second cohort, more than 100 samples were obtained from the primary tumors and metastatic sites of 26 patients and forwarded to targeted sequencing of a limited number of genes and not the whole exome [7]. They found discrepant pathogenic alterations between primary tumors and paired metastatic lesions in 45% of the patients. Among alterations in receptor tyrosine kinases, 9 of 12 cases (75%) were discordant across all matched samples [7]. Von Loga et al. [8] recently studied four MSI GCs by multiregional sequencing and found an extreme intratumoral heterogeneity as well as evidence of parallel evolution in this special, but rare subtype of GC. As shown here, MSI GC may not be representative for more prevalent types of GC.

Thus, our study extends previous studies, e.g., on gastric adenomas [46], particularly by including also more prevalent types of GC. We show that the lateral expansion of GC is associated with substantial genetic variability, and lymph node metastases may stem synchronously from different areas of the primary tumor. Single-sample analysis of the primary tumor as well as of lymph node metastases may miss between 53.2 and 91.3% of the mutations present in a single GC. In a subset of genes the genotype is also variable and single-sample analysis may miss signs of parallel evolution and hence underestimate the importance of genes/pathways. In this respect, it is interesting to note that a comparison of the prevalences of gene mutations obtained by multiregional sequencing provides figures quite different from those obtained by single-sample analysis (e.g., 8% SMAD4 mutations in the TCGA data set, 15% in a series of 551 esophageal adenocarcinomas, and 33% in our dataset; Additional file 2: Table S13) [3, 43]. These differences illustrate the risk of mis-interpreting the significance of individual genes and pathways in GC biology when findings are based only on single-sample analyses. Thus, multiregional sequencing also provides insights into cancer biology, which are missed by single-sample analysis.

This, for instance, applies to the classification of clonality. The presence of a particular gene mutation in every tissue sample of a given tumor does not necessarily represent clonality and has been referred to as “clonal illusion” [35]. Our bioinformatic approach demonstrates that only a minority of the homogeneously distributed mutations could be classified with reasonable certainty as truly clonal (Table 1) and that the assessment of clonality is a function of the existing mutational landscape of the tumor, which shows interindividual variability, and the number of samples available: in six cases, a correct assessment of clonality would require the analysis of additional samples for an accurate annotation, particularly in highly unbalanced tumors (Fig. 3). Thus, even multiregional sequencing carries a risk of mis-interpreting clonality.

Cancer is an evolutionary disease and four models are discussed in this context, i.e., the “sequential,” “branched,” “neutral,” and “punctuated” model [41]. Here we tested the neutral model and showed that the compatibility with neutrality was variable between different samples from the same tumor contradicting the concept of a single mode of expansion for the entire tumor. This observation may also lend support to the hypothesis that different modes of evolution might be operating within a single tumor. However, it has to be kept in mind that even multiregional sequencing only provides a snapshot of a highly dynamic disease process and provides no information regarding the temporal and microenvironmental constraints. Intratumoral heterogeneity resulting from somatic evolution might be attributable to ongoing genetic and heritable epigenetic alterations and selection might be operative in certain but not all microenvironments, i.e., different histoanatomical layers of the stomach wall, within lymph nodes or a metabolic environment mediated by chaotic angiogenesis, immune response, and various other factors [47]. In this respect, the discovery of a subclonal amplification of *CD274* (PD-L1), which was associated with a strong expression of PD-L1 (Fig. 2), in a chemotherapy-naïve case is highly intriguing and shows that tumor subclones can acquire distinctive immune evasion capabilities in the absence of preceding perioperative chemotherapy.

Somatic evolution of GC has several major clinical implications regarding the assessment of putative subclonal genetic events as well as tissue-based precision medicine. While clonal events might lead to cancer initiation, at later disease stages the initiating genetic lesions may no longer ensure cell survival or might have little influence on patient prognosis [47]. In a preceding study, we were unable to relate p53-alterations in GC with patient outcome [30]. By generating multiregional trees, we identified subclonal *SMAD4* mutations in four cases of the discovery cohort. Subsequent validation of the biological significance of the SMAD4 expression in a large Caucasian patient population provided strong evidence of the prognostic value of SMAD4 loss. It turned out to be an independent prognosticator of OS and TSS at least for cytoplasmic expression of SMAD4. SMAD4 shuttles between the cytoplasm and nucleus. Immunostaining usually is cytoplasmic, while nuclear staining can also be detected in non-mutant cases, as also shown here. *SMAD4* mutations affect both, cytoplasmic and nuclear staining [48]; however, only loss of cytoplasmic immunostaining was an independent prognosticator of patient survival in our cohort. Interestingly, Kaplan-Meier analyses using only cases with a known *TP53* genotype showed that patients with any loss of nuclear SMAD4 expression had a lower median OS and TSS (Additional file 2: Figure S5). These data suggest that loss of nuclear staining is also prognostically relevant in *TP53* mutant cases. Further studies are warranted to clarify the distinctive role of cytoplasmic and nuclear SMAD expression in GC biology.

To some extent, similar findings on the prognostic significance of SMAD4 in GC were made in three Asian cohorts [49–51] and a two Caucasian cohorts [43, 52], however, with some differences and some major limitations: The number clinicopathological patient characteristics was limited in all preceding studies and none included resection, *H. pylori*, EBV, MSI, and p53 status [43, 49–52]. Resection status and MSI are important predictors of patient survival and we are the first to demonstrate that cytoplasmic SMAD4 loss remains an independent prognosticator of OS and TSS even when the resection status and MSI are included in multivariate analyses. The majority of the preceding studies tested not for false discovery rates [49–52]. In addition, the overall prognosis of GC in Asian patients is substantially different from GC in Caucasian patients and findings made in Asian patient populations cannot be translated untested into Caucasian cohorts [53]. One of two studies exploring SMAD4 in Caucasian patients was limited to a series of 151 cases without multivariate analyses [52]. The second study on Caucasian patients correlated SMAD4 genotype with patient outcome and did not assess the expression pattern [43]. Thus, our study is the first extended exploration of the tumor biological significance of SMAD4 in a large and well characterized Caucasian patient population confirming the independent prognostic significance of SMAD4 expression in GC.

The subclonal alteration of *SMAD4* points towards another highly interesting issue: it was almost exclusively found in *TP53* mutant GCs. Tumor-initiating genetic events may influence subsequent evolutionary trajectories and may lead to parallel evolution, in which the fitness state of specific subclones depends on mutations in the same gene (*SMAD4*) or pathway (e.g., TGFβ signaling pathway). While these subclonal mutations could be missed by the analysis of a single bulk tissue sample (as shown here), identification of the clonal ground state may provide highly valuable information with regard to the future (most likely) subclonal alterations necessary for tumor progression. Tumor progression could depend on epistatic genetic interactions in which the functional effects of genetic mutations are determined by their temporal order leading to evolutionary trajectories [54]. Evolutionary trajectories might also partially explain the intratumoral heterogeneity: 369 genes harbored two to four different non-synonymous mutations in the same patient. Some might be irrelevant passenger mutations (according to the neutral model), but some might represent other evolutionary trajectories (i.e., deterministic temporal order of mutations) and the analysis of additional patient cohorts is urgently needed to identify further trajectories in GC [43]. Each individual subclonal mutation merits in-depth validation studies to explore its putative role in cancer biology since subclonal mutations could be neutral or clinically relevant, as has been shown here concerning SMAD4.

With regard to *TP53* mutant cases, we also noted subclonal alterations of members of the SWI/SNF pathway. This finding is in line with our recent study in which loss of ARID1A correlated inversely with MSI and EBV status in GC [55], molecular subtypes of GC usually showing a low prevalence of *TP53* mutations [3]. Thus, alterations of the SWI/SNF pathway may point to an additional trajectory in *TP53* mutant GCs. Parallel somatic evolution might also explain the intratumoral heterogeneity of *PIK3CA* mutations in EBV-associated GCs [4]. Thus, the four molecular subtypes of GC might be extended by the identification of subtype-specific evolutionary trajectories and further studies on this topic are urgently needed.

Summing up, in a reductionist view, we show that the lateral expansion of GC is associated with substantial genetic variability, and lymph node metastases may stem synchronously from different areas of the primary tumor. We confirm that single-sample analysis, including lymph node metastases, may miss between 53.2 and 91.3% of the mutations present in GC. This also applies to CNVs of genes involved in immune evasion strategies, e.g., PD-L1. We found evidence of parallel evolution, which applies to single genes as well as to pathways. The assessment of clonality depends on the extent of interindividual variability of intratumoral heterogeneity, and hence the number of samples studied. Thus, multiregional sequencing and the generation of multiregional trees also carry a risk of mis-interpreting clonality. Multiple modes of evolution (e.g., neutral vs. non-neutral) could be operating concurrently in GC. We found evidence of biologically relevant evolutionary trajectories in GC, which is probably a driver of parallel evolution: cancer progression in *TP53* mutant GCs is linked to subsequent (putative subclonal) alterations in the TGFβ and SWI/SNF pathway.

## Conclusions

Looking into the future of precision medicine and based on the findings of our study, a combined approach using the identification of druggable targets by comprehensive molecular analysis, unveiling the mode of tumor expansion (neutral vs. non-neutral) and the discovery of distinct evolutionary trajectories may aid in finding the best treatment for a particular tumor and also ultimately may lead to an evolutionary classification of GC.

## Supplementary Information

**Additional file 1: **Figure S1. Figure S1. Tissue sampling procedure of the discovery cohort. Figure S2. Variant allele frequency (VAF) histograms generated from the discovery cohort. Figure S3. Maximum parsimony multiregional trees. Figure S4. Kaplan-Meier curves of the validation cohort (SMAD4). Figure S5. Kaplan-Meier curves of a subset of the validation cohort with known *TP53*-genotype.

**Additional file 2:.** Table S1. Table S1. List of purity- and ploidy status of the discovery cohort. Table S2. Sequencing statistics. Table S3. Primer sequences used for Sanger sequencing, pyrosequencing and ddPCR™. Table S4. Sanger sequencing, pyrosequencing and ddPCR™ were used to validate single nucleotide variations detected by whole-exome sequencing. Table S5. List of non-synonymous mutations found in the discovery cohort. Table S6. List of synonymous mutations found in the discovery cohort. Table S7. List of genes with non-synonymous mutations present in the primary tumor of case #5 and its lymph node metastases. Table S8. Distribution of the copy number variants (CNVs), i.e., homozygous or heterozygous deletion and amplification among the 48 tumor samples of the discovery cohort. Table S9. List of cancer cell fraction (CCF) non-synonymous mutations. Table S10. List of genes with ≥2 non-synonymous mutations per case. Table S11. Pathway analysis including copy number variation on the discovery cohort. Table S12. Multivariate analysis. Table S13. Comparison of the prevalence of the single nucleotide variations discovered in our test cohort with published data on gastric cancer [3, 5, 7, 56]. Table S14. List of genes with non-synonymous mutations present in ≥2 cases of the discovery cohort.

**Additional file 3:.** Results S1

## Data Availability

All data generated or analyzed during this study from all gastric cancer patients are included in this published article and its supplementary files. Raw sequencing data are available at the European Genome Archive (EGAS00001004525) https://ega-archive.org/studies/EGAS00001004525 [32].

## Abbreviations

AF: Allele frequency
CIN: Chromosomal instability
CNV: Copy number variation
EBV: Epstein-Barr virus
GC: Gastric cancer
GS: Genomic stability
MSI: Microsatellite instability
SNV: Single-nucleotide variations
VAF: Variant allele frequency
