# Supplementary material for: Multiscale heterogeneity in gastric adenocarcinoma evolution is an obstacle to precision medicine
Source: Genome Med. 2021 Nov 8;13:177. doi: 10.1186/s13073-021-00975-y (PMC8576943; doi:10.1186/s13073-021-00975-y)
Supplement: Supplementary file 1 — Additional file 1: Figure S1. Figure S1. Tissue sampling procedure of the discovery cohort. Figure S2. Variant allele frequency (VAF) histograms generated from the discovery cohort. Figure S3. Maximum parsimony multiregional trees. Figure S4. Kaplan-Meier curves of the validation cohort (SMAD4). Figure S5. Kaplan-Meier curves of a subset of the validation cohort with known TP53-genotype. [file 13073_2021_975_MOESM1_ESM.pptx]

## Slide 1
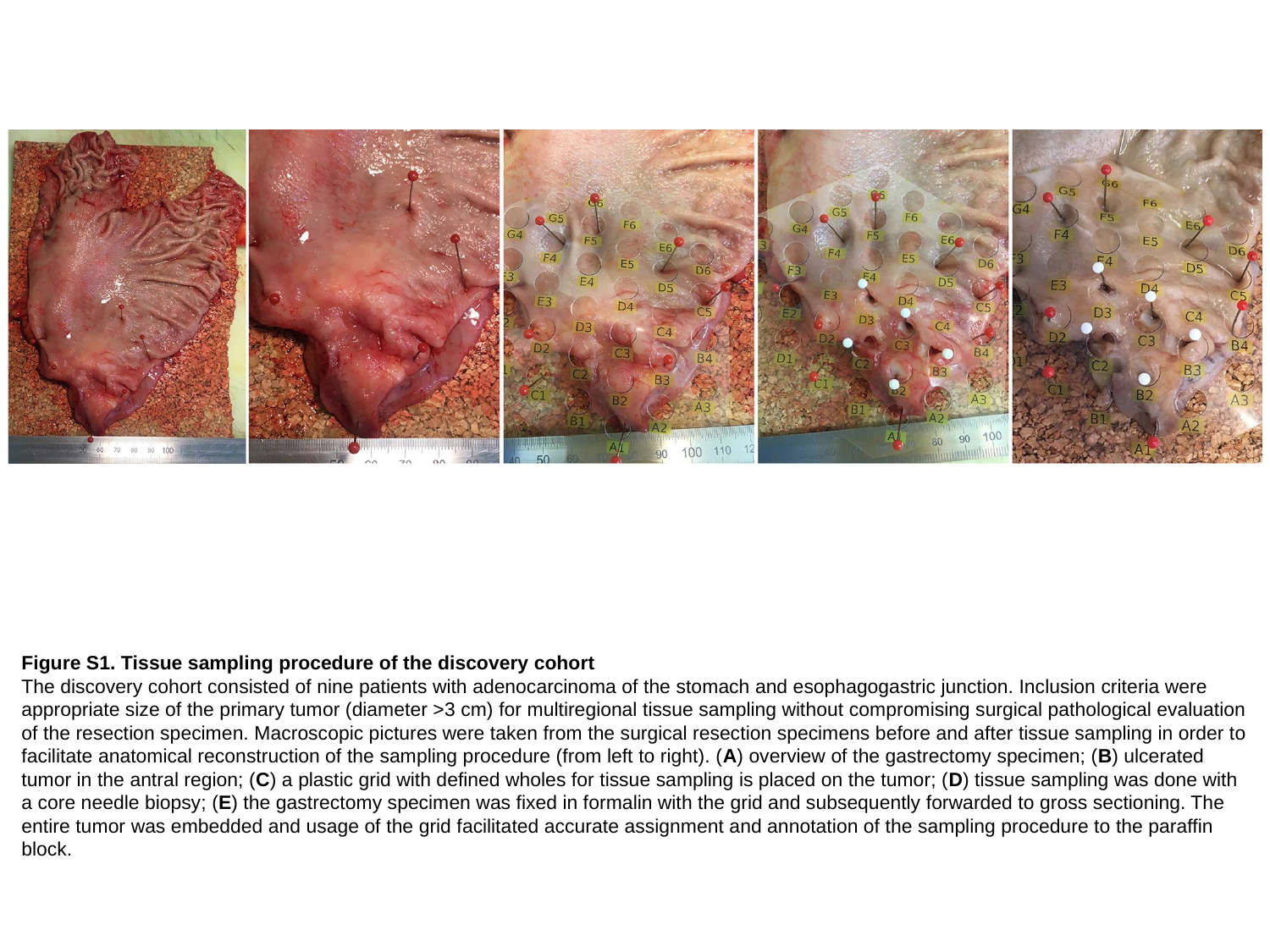

Figure S1. Tissue sampling procedure of the discovery cohort
The discovery cohort consisted of nine patients with adenocarcinoma of the stomach and esophagogastric junction. Inclusion criteria were appropriate size of the primary tumor (diameter >3 cm) for multiregional tissue sampling without compromising surgical pathological evaluation of the resection specimen. Macroscopic pictures were taken from the surgical resection specimens before and after tissue sampling in order to facilitate anatomical reconstruction of the sampling procedure (from left to right). (A) overview of the gastrectomy specimen; (B) ulcerated tumor in the antral region; (C) a plastic grid with defined wholes for tissue sampling is placed on the tumor; (D) tissue sampling was done with a core needle biopsy; (E) the gastrectomy specimen was fixed in formalin with the grid and subsequently forwarded to gross sectioning. The entire tumor was embedded and usage of the grid facilitated accurate assignment and annotation of the sampling procedure to the paraffin block.

## Slide 2
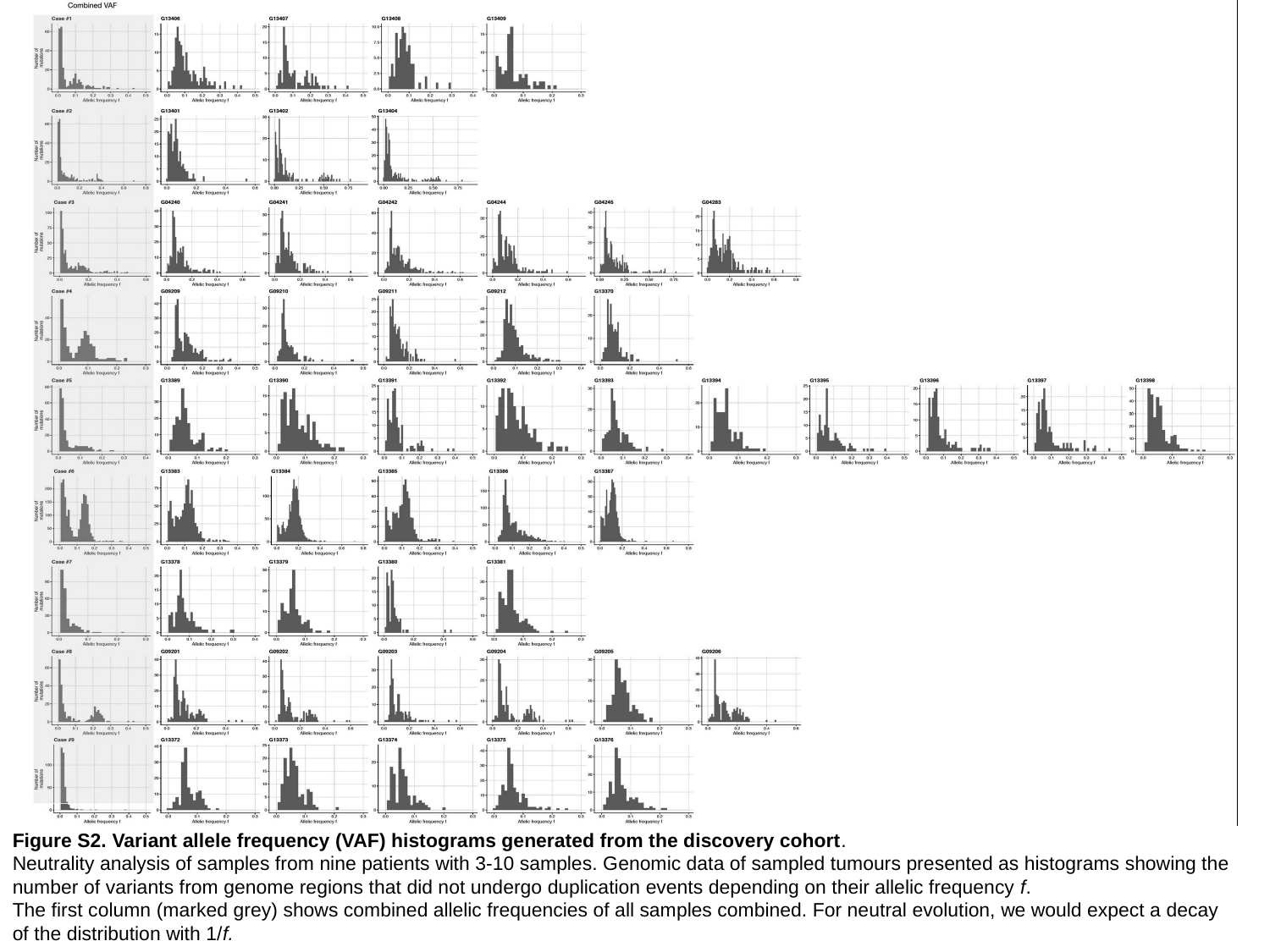

Figure S2. Variant allele frequency (VAF) histograms generated from the discovery cohort.
Neutrality analysis of samples from nine patients with 3-10 samples. Genomic data of sampled tumours presented as histograms showing the number of variants from genome regions that did not undergo duplication events depending on their allelic frequency f.
The first column (marked grey) shows combined allelic frequencies of all samples combined. For neutral evolution, we would expect a decay of the distribution with 1/f.

## Slide 3
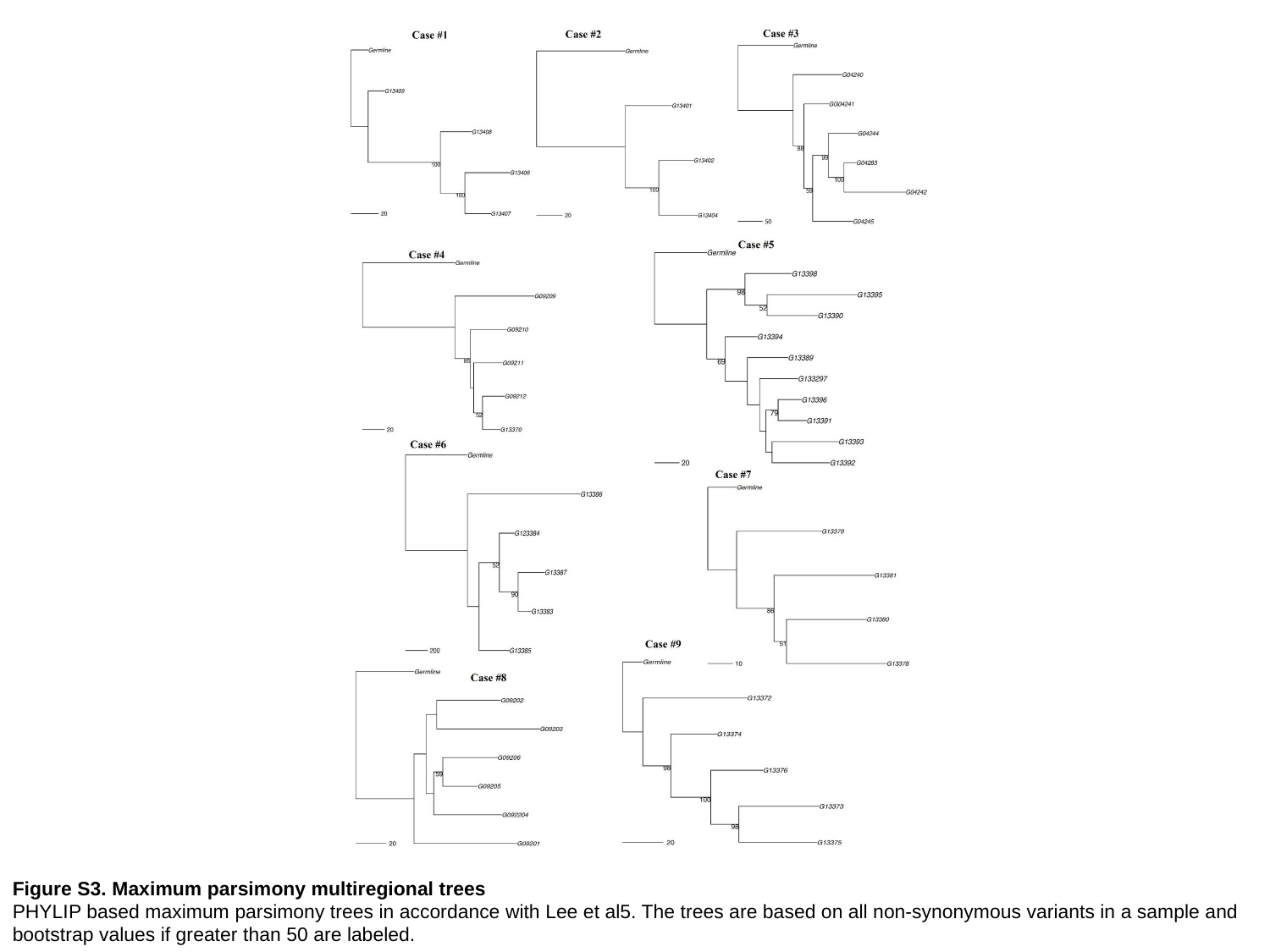

Figure S3. Maximum parsimony multiregional trees
PHYLIP based maximum parsimony trees in accordance with Lee et al5. The trees are based on all non-synonymous variants in a sample and bootstrap values if greater than 50 are labeled.

## Slide 4
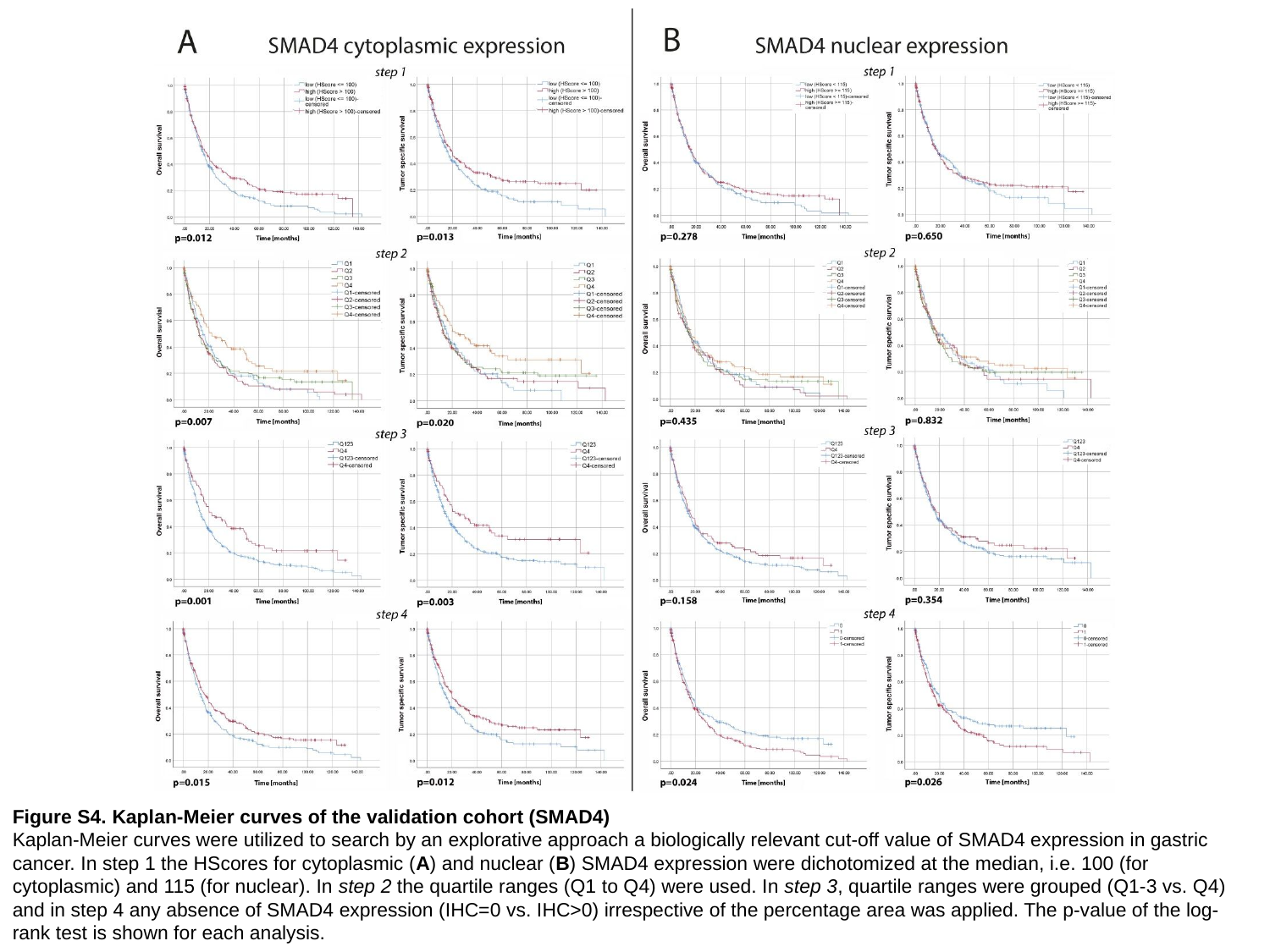

Figure S4. Kaplan-Meier curves of the validation cohort (SMAD4)
Kaplan-Meier curves were utilized to search by an explorative approach a biologically relevant cut-off value of SMAD4 expression in gastric cancer. In step 1 the HScores for cytoplasmic (A) and nuclear (B) SMAD4 expression were dichotomized at the median, i.e. 100 (for cytoplasmic) and 115 (for nuclear). In step 2 the quartile ranges (Q1 to Q4) were used. In step 3, quartile ranges were grouped (Q1-3 vs. Q4) and in step 4 any absence of SMAD4 expression (IHC=0 vs. IHC>0) irrespective of the percentage area was applied. The p-value of the log-rank test is shown for each analysis.

## Slide 5
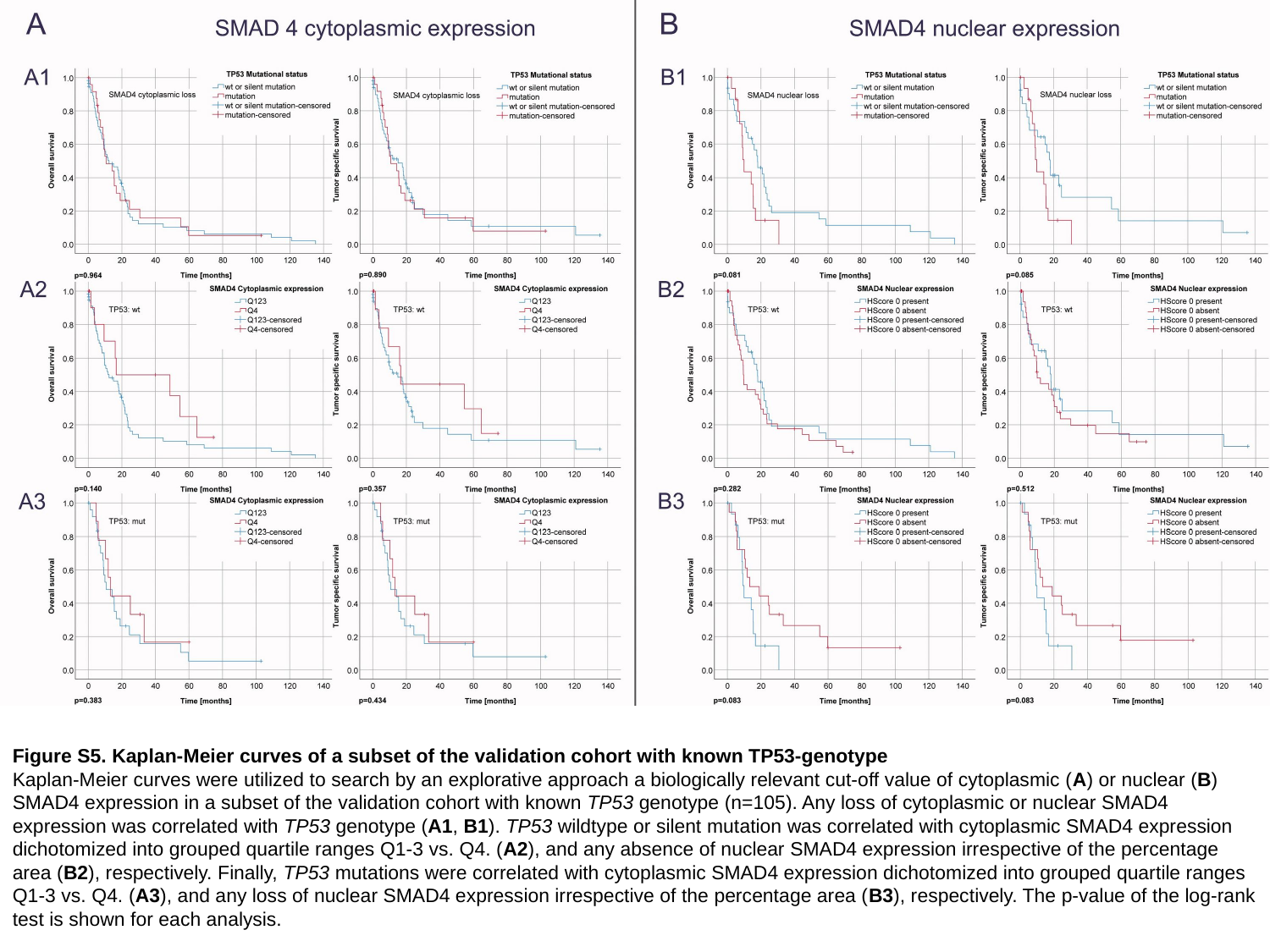

Figure S5. Kaplan-Meier curves of a subset of the validation cohort with known TP53-genotype
Kaplan-Meier curves were utilized to search by an explorative approach a biologically relevant cut-off value of cytoplasmic (A) or nuclear (B) SMAD4 expression in a subset of the validation cohort with known TP53 genotype (n=105). Any loss of cytoplasmic or nuclear SMAD4 expression was correlated with TP53 genotype (A1, B1). TP53 wildtype or silent mutation was correlated with cytoplasmic SMAD4 expression dichotomized into grouped quartile ranges Q1-3 vs. Q4. (A2), and any absence of nuclear SMAD4 expression irrespective of the percentage area (B2), respectively. Finally, TP53 mutations were correlated with cytoplasmic SMAD4 expression dichotomized into grouped quartile ranges Q1-3 vs. Q4. (A3), and any loss of nuclear SMAD4 expression irrespective of the percentage area (B3), respectively. The p-value of the log-rank test is shown for each analysis.
