# Supplementary material for: Multiscale heterogeneity in gastric adenocarcinoma evolution is an obstacle to precision medicine
Source: Genome Med. 2021 Nov 8;13:177. doi: 10.1186/s13073-021-00975-y (PMC8576943; doi:10.1186/s13073-021-00975-y)
Supplement: Supplementary file 3 — Additional file 3:. Results S1 [file 13073_2021_975_MOESM3_ESM.docx]

**Additional File 3**

Multiscale heterogeneity in gastric adenocarcinoma evolution is an obstacle to precision medicine

Christoph Röcken^1^, Anu Amallraja^2^, Christine Halske^1^,

Luka Opasic^3^, Arne Traulsen^3^, Hans-Michael Behrens^1^, Sandra Krüger^1^, Anne Liu^1^,

Jochen Haag^1^, Jan-Hendrik Egberts^4^, Philip Rosenstiel^4^, Tobias Meißner^2^

^1^Dept. of Pathology, University Hospital Schleswig-Holstein (UKSH), Campus Kiel, Germany

^2^Dept. of Molecular and Experimental Medicine, Avera Cancer Institute, Sioux Falls, USA

^3^Dept. of Evolutionary Theory, Max Planck Institute for Evolutionary Biology,

Plön, Germany

^4^Dept. of General Surgery, Visceral, Thoracic, Transplantation and Pediatric Surgery, University Hospital Schleswig-Holstein (UKSH), Campus Kiel, Germany

^4^Institute for Clinical Molecular Biology, Christian-Albrechts-University, 24105 Kiel, Germany

**Supplemental Results**

*Sequencing data of the discovery cohort*

The median sequencing coverage of the discovery cohort was 163x for tumor samples and 170x for non-neoplastic mucosa (Additional file 2: Table S2). Median tumor purity (computationally assessed by Sequenza) was 33% (12%-98%) (Additional file 2: Table S1). Raw sequencing data were deposited at the European Genome Archive (EGAS00001004525)[32]. The results of the whole exome sequencing of the discovery cohort are summarized in Additional file 2: Table S5-S10 and S14.

*Validation analyses using Sanger sequencing, pyrosequencing and ddPCR™*

Single nucleotide variations (SNV) detected by whole exome sequencing were validated independently in the test cohort using Sanger sequencing, pyrosequencing or ddPCR™ (Additional file 2: Table S4).

In total, 179 analyses were done and SNVs of the following genes could be validated independently: *AKT1* (5 of 5 tissue samples), *BRCA1* (5 of 9), *BRCA2* (5 of 5), *CDH1* (10 of 10), *CTNNB1* (6 of 6), *FLT4* (6 of 6), *IKBKB* (11 of 11), *IKZF3* (4 of 4), *KRAS* (5 of 5), *LRP1B* (12 of 12), *MAP2K4* (5 of 5), *MCM8* (6 of 6), *MLH1* (4 of 4), *MUTYH* (5 of 5), *PAX5* (5 of 5), *PIK3CA* (4 of 5), *POLE* (4 of 5), *RNF43* (4 of 4), *SMAD4* (26 of 26), and *TP53* (8 of 9)(Additional file 2: Table S4). The SNVs of the following genes could not be validated either due to inability of designing an appropriate assay or due to an allele frequency below detection limit: *ARID1A*, *ARID1B*, *ASXL3*, *CLOCK*, *PRRC2A* and *TP53BP1* (Additional file 2: Table S4).

*Somatic signatures*

We analyzed the observed somatic signatures in their 5’ and 3’ base context thus resulting in 96 possible mutation types [56]. Based on the exome data, we retrieved the individual mutational signature from each tumor sample in relation to the non-tumorous tissue. We found that the somatic signatures of GCs were consistent with previous reports: signature 1 was found in 48 tumor samples (100%), signature 15 in 48 (100%), signature 18 in 29 (60.4%), signature 10 in 3 (6.3%) and signature 29 in 2 samples (4.2%) [56].

*Comparison with published sequencing data*

We compared our non-synonymous mutations with published data (Additional file 2: Table S13). Previously reported prevalent non-synonymous mutations were also present in our cohort, albeit with deviations regarding their individual prevalence [3, 5, 7, 55], which is probably due to patient selection as well as the number of samples analyzed per patient. Mutations found in 6 (67%) patients of our series affected *LRP1B*; in 5 (56%) patients *TP53*; in 4 (44%) patients *CDKN2A*, *KMT2D* and *RNF43*; in 3 (33%) patients *ABCA10*, *ARID1B*, *BCL2*, *BNC2*, *MACF1*; *MYC*, *NRG1*, *PTEN* and *SMAD4;* and in 2 (22%) patients *BIRC6*, *CCNE1*, *CTNNB1*, *ELF3*, *ERBB2*, *FAM135B*, *GPC5*, *KMT2C*, *MLH1*, *MTOR*, *NF1*, *PIK3CA*, *POLE*, *RIMS2*, *ROS1*, *SMARCA4* and *TGFBR2* (Additional file 2: Table S13). A total of 131 genes (0.03% of all affected genes) showed homogeneously distributed non-synonymous mutations in ≥2 patients (Additional file 2: Table S14).

*SMAD4 expression in the validation cohort*

SMAD4 expression was studied using whole tissue sections and a validation cohort of 487 GCs. The clinicopathological patient characteristics are summarized in Table 2.

Weak nuclear immunostaining of tumor cells was found in 430 (88.3%), a moderate in 382 (78.4%) and a strong in 84 (17.2%) GCs. Immunonegative tumor cells were found in 279 GCs (57.3%) cases. Weak cytoplasmic immunostaining of tumor cells was found in 450 (92.4%), a moderate in 307 (63.0%) and a strong in 34 (7.0%) GCs. A complete loss of cytoplasmic immunostaining of tumor cells was found in 255 GCs (52.4%). The percentage area of the four immunostaining categories ranged from 0 to 100% for both, nuclear and cytoplasmic SMAD4 expression, and the combination of the staining categories in each individual case varied. The median HScore was 115 (range 0-300) for nuclear and 100 (range 0-245) for cytoplasmic SMAD4 expression. 22 (4.5%) GCs were completely devoid of any nuclear and 27 (5.5%) for cytoplasmic SMAD4 expression. Nuclear and cytoplasmic immunostaining correlated highly significantly with each other (Pearson's correlation coefficient; p<0.001; data not shown). The expression of SMAD4 was heterogeneous including “grey-scale” as well as “black-and-white”-immunostaining patterns (Figure 2E). The latter showed areas of complete loss of SMAD4 expression clearly demarcated from areas with retained SMAD4 expression (see Figure 2E). A black-and-white expression pattern was found in 181 (37.2%) GCs for nuclear immunostaining and in 170 (34.9%) GCs for cytoplasmic immunostaining.

To explore the putative biological significance of SMAD4 in GC we correlated its expression pattern with various clinicopathological patient characteristics. However, in view of a heterogeneous expression and since we did not know *a priori*, which “cut off” value, i.e., degree of decreased SMDA4-expression and/or percentage area of SMAD4-loss might be biological relevant we applied a stepwise explorative approach using patient survival as surrogate marker for biological significance: In the first step (Additional file 1: Figure S4A & S4B; *step 1*) we dichotomized the cohort at the median HScores, i.e., 100 (for cytoplasmic) and 115 (for nuclear), in *step 2* we used the quartile ranges (Q1 to Q4), in *step 3* we grouped the quartile ranges (Q1-3 vs. Q4) and finally we explored any absence of SMAD4 expression (IHC=0 vs. IHC>0; *step 4*) irrespective of the percentage area. We used the log-rank test to find an appropriate cut-off value. Finally, we used the grouped quartile ranges (Q1-3 vs. Q4; *step 3*) of cytoplasmic SMAD4 expression and the absence of any nuclear SMAD4 expression (IHC=0; *step 4*) for the correlation with diverse clinicopathological patient characteristics.

Following this dichotomization (Table 2), cytoplasmic SMAD4 expression correlated with local tumor growth (T category) as well as overall and tumor-specific survival (all significant after correction for multiple testing). Nuclear SMAD4 expression was lower in GCs of the distal stomach, diffuse and unclassifiable GCs (according to Lauren), and had a worse outcome, However, none of these correlations proved significant after correction for multiple testing.
